# Integrated single cell data analysis reveals cell specific networks and novel coactivation markers

**DOI:** 10.1186/s12918-016-0370-4

**Published:** 2016-12-05

**Authors:** Shila Ghazanfar, Adam J. Bisogni, John T. Ormerod, David M. Lin, Jean Y. H. Yang

**Affiliations:** 1 School of Mathematics and Statistics, The University of Sydney, Eastern Avenue, Camperdown, NSW, 2006 Australia; 2 Department of Biomedical Sciences, Cornell University, Ithaca, NY, 14853 USA; 3 ARC Centre of Excellence for Mathematical & Statistical Frontiers, University of Melbourne, Parkville VIC, 3010 Australia

**Keywords:** Single-cell transcriptomics, RNA-sequencing, Mixture modelling, ScRNA-Seq, Olfactory sensory neuron, Neuron

## Abstract

**Background:**

Large scale single cell transcriptome profiling has exploded in recent years and has enabled unprecedented insight into the behavior of individual cells. Identifying genes with high levels of expression using data from single cell RNA sequencing can be useful to characterize very active genes and cells in which this occurs. In particular single cell RNA-Seq allows for cell-specific characterization of high gene expression, as well as gene coexpression.

**Results:**

We offer a versatile modeling framework to identify transcriptional states as well as structures of coactivation for different neuronal cell types across multiple datasets. We employed a gamma-normal mixture model to identify active gene expression across cells, and used these to characterize markers for olfactory sensory neuron cell maturity, and to build cell-specific coactivation networks. We found that combined analysis of multiple datasets results in more known maturity markers being identified, as well as pointing towards some novel genes that may be involved in neuronal maturation. We also observed that the cell-specific coactivation networks of mature neurons tended to have a higher centralization network measure than immature neurons.

**Conclusion:**

Integration of multiple datasets promises to bring about more statistical power to identify genes and patterns of interest. We found that transforming the data into active and inactive gene states allowed for more direct comparison of datasets, leading to identification of maturity marker genes and cell-specific network observations, taking into account the unique characteristics of single cell transcriptomics data.

**Electronic supplementary material:**

The online version of this article (doi:10.1186/s12918-016-0370-4) contains supplementary material, which is available to authorized users.

## Background

High throughput transcriptome profiling of single cells has exploded in recent years in the areas of biomedical and basic science research. Single cell RNA-Seq (scRNA-Seq) has been employed to study many types of cells in a number of organisms, including stem cells, cancer cells and neurons in mouse and human [1]. This technology has enabled both small-scale interrogations (16 cells [2]) to very large scale profiling studies (44,808 cells [3]) on a transcriptome level. Analysis approaches have aimed to characterize cell heterogeneity, and to identify subtypes using techniques such as dimension reduction and clustering. Other possible analyses include differential expression, and interrogating more specific questions. For instance, a long standing hypothesis has been that olfactory sensory neurons (OSNs) in mice express only one odorant receptor gene, termed the ‘one-neuron-one-receptor’ rule, which was able to be tested in single mature and immature OSNs through scRNA-Seq, and the authors found that immature neurons can transiently express multiple odorant receptor genes [4] while mature neurons primarily express one odorant receptor gene. A number of tools and approaches have emerged recently offering extensive pipelines from raw reads to analysis results [5–7], and others that focus on particular aspects of a typical scRNA-Seq analysis, such as clustering [8, 9] and differential expression analysis [10].

Some statistical challenges associated with scRNA-Seq are unique compared to typical RNA-Seq of bulk cell populations. While shared challenges such as normalization, accurate modeling of counts and cross platform comparisons exist, these may be exacerbated or manifest differently in the presence of features unique to scRNA-Seq data. The most immediate characteristic of single cell gene expression count matrices are that there is an abundance of zeros, i.e. genes with no read counts [10], that persist even after transformations such as counts per million (CPM) or reads per kilobase per million reads (RPKM). Furthermore, the proportion of zeros across genes appears to be related to the depth of sequencing performed, contributing to the challenge of appropriately comparing between multiple datasets with different levels of read depths achieved.

Another key aspect of scRNA-Seq data is the apparent bimodality of non-zero expression values [11–13]. As this phenomenon is also observed in other single-cell gene expression measurement methods such as fluorescence in situ hybridization (FISH) [11], it is believed that this phenomenon is not attributed to technical artifacts stemming from the scRNA-Seq experiments. Rather, examining the distribution of gene expression measurements of a given gene over many cells can uncover three distinct transcriptional states: no expression, characterized by no observed read counts; low expression, where RNA is present at a low level and possibly undergoing degradation; and high expression, where RNA may have been produced through a ‘bursting’ process [14]. Existing approaches for classifying cells into a low or high expression state are few, including imposing a strict threshold value, and fitting Gaussian mixture models [15].

To this end, in this manuscript we offer a versatile modeling framework to identify transcriptional states as well as structures of coactivation for different neuronal cell types across multiple datasets. This framework includes (1) a gamma-normal mixture modeling approach to classify each gene into no, low or high expression within each cell; (2) the identification of coactivation networks within each cell and (3) creation of a uniqueness metric to identify cell type specific genes across multiple scRNA-Seq datasets. Furthermore, we focused on three specific datasets that studied olfactory sensory neurons, and discovered that the topology of coactivation networks of each cell changes as the olfactory sensory neuron cells mature. This work enables discovery of biologically meaningful genes through combined analysis of coactivation with genes known to be related to neuron maturity.

## Methods

### Data collection and preprocessing

A set of nine single cell RNA-Seq datasets were curated (Table 1), all measuring transcriptomes of various neuronal cell populations in mice, with varying numbers of cells, sequencing strategies, and overall read depths. Raw sequencing reads were downloaded from the Gene Expression Omnibus (GEO), the Sequence Read Archive (SRA) or the European Nucleotide Archive (ENA). Fastq files were each mapped to the mm10 reference genome using STAR RNA-Seq aligner [16] with default parameters. The resulting mapped read files were then converted to bam, sorted and indexed using Samtools [17], and read counts for a total of 38806 genes were obtained using HTSeq-count [18] under the mode ‘union’ with other default parameters. Read counts for multiple runs belonging to the same cell were added together, resulting in a raw count matrix for 38806 genes and 6377 cells. The data matrix was further transformed by calculating counts per million mapped reads (CPM) and taking the shifted log (log2CPM), i.e. $y_{ij} = \log _{2}(1 + 10^{6} r_{ij}/\sum _{k}r_{kj})$, where *r*_*ij*_ are the raw read counts and *y*_*ij*_ the transformed counts for gene *i* and cell *j*. Following this, we fitted gamma-normal mixture models per gene per dataset, initially removing cells with zero log2CPM values, as described in the next section.

**Table 1:** Description of nine murine neuronal single-cell RNA-Seq datasets. DRG - dorsal root ganglion

Author	GEO/SRA Accession	Number of Cells	Cell Type(s)	Read Length	Median Read Depth
Fuzik et al. [37]	GSE70844	83	Excitatory pyramidal and inhibitory neurons	51	391,449
Hanchate et al. [25]	GSE75413	93	Olfactory sensory neurons	98	3,352,691
Li et al. [23]	GSE63576	209	Somatosensory DRG neurons	200	18,300,045
Lovatt et al. [24]	GSE52525	28	Mixed cultures of dispersed braincells, hippocampal pyramidal neurons	202	17,727,180
Saraiva et al. [15]	PRJEB4014, PRJEB8101, PRJEB4461	264	Olfactory sensory neurons	200	1,570,234
Tan et al. [4]	SRP065920	143	Olfactory sensory neurons	100	936,016
Tasic et al. [38]	GSE71585	1,809	Cortical cells	89	2,350,114
Usoskin et al. [39]	GSE59739	864	Lumbar DRG neurons	40	86,588
Zeisel et al. [40]	GSE60361	3,005	Somatosensory and hippocampal C1 neurons	52	496,431
Total		6498			

### Gamma-normal mixture modeling

To model the distribution of gene expression values, we considered a gamma-normal mixture model. The gamma distribution is fairly flexible with two parameters, and takes non-negative values. We observe that scRNA-Seq gene expression values on log2CPM scale also take non-negative values and thus this distribution may be suitable. We may be able to use a simpler distribution with similar properties, such as the exponential distribution, however this does not appear to be as flexible as desired. As well as this, the normal distribution is a suitable candidate for the second component of the mixture model as it is fairly well characterized.

Before continuing with fitting the gamma-normal mixture model, we remove the zeroes from the data, as data arising from scRNA-Seq exhibits an extremely high proportion of genes and cells with zero counts, resulting in ill-fitting model parameters.

The remainder of this section derives the expectation maximization (EM) algorithm for fitting the gamma-normal mixture model. We assume that for a given gene, the non-zero expression values can be described by a mixture of gamma and normal distributions, where the gamma component corresponds to lowly expressed cells and the normal components corresponds to the highly expressed cells. Let **x**=(*x*_1_,*x*_2_,…,*x*_*n*_) be a vector of non-zero log2CPM expression values for a given gene. The density functions for the gamma and normal component are

$$\begin{aligned} f(w,\alpha,\beta) &= \frac{\beta^{\alpha}}{\Gamma(\alpha)}w^{\alpha-1}e^{-\beta w}, \quad \text{and} \\ f\left(w,\mu,\sigma^{2}\right) &= \frac{1}{\sigma \sqrt{2 \pi}}e^{-\frac{(w-\mu)^{2}}{2\sigma^{2}}} \end{aligned} $$

respectively. Let **y**=(*y*_1_,*y*_2_,…,*y*_*n*_) be a binary vector indicating membership of each cell in the normal component of the mixture. We assume that *y*_*i*_ is generated from an independent Bernoulli distribution with probability of success *ρ*,*y*_*i*_∼*B*(1,*ρ*) for *i*=1,2,…,*n*. Thus the density function for *x*_*i*_ is

$$\begin{aligned} f(x_{i},\alpha,\beta,\mu,\sigma^{2},\rho)& = (1-\rho)\frac{\beta^{\alpha}}{\Gamma(\alpha)}x_{i}^{\alpha-1}e^{-\beta x_{i}}\\&\quad+ \rho \frac{1}{\sigma \sqrt{2 \pi}}e^{-\frac{(x_{i}-\mu)^{2}}{2\sigma^{2}}}. \end{aligned} $$

The corresponding complete log-likelihood is

$$\begin{aligned} &\ell(\mathbf{x},\mathbf{y},\alpha,\beta,\mu,\sigma^{2},\rho) \\ &=\sum_{i=1}^{n} \left[(1-y_{i})\left(\alpha \log(\beta) - \log \Gamma(\alpha) \right. \right.\\ &\qquad\left.+ (\alpha - 1) \log(x_{i}) - \beta x_{i} \right)\\ &\qquad+ y_{i} \left(- \frac{1}{2} \log\left(2 \pi \sigma^{2}\right) - \frac{\left(x_{i}-\mu\right)^{2}}{2\sigma^{2}} \right) + y_{i} \log \rho \\&\qquad\left.+ (1-y_{i})\log(1-\rho) \right]. \end{aligned} $$

Let $z_{i} = \mathbb {E}(y_{i} | \text {rest}), i = 1,2,\ldots,n$ be the expectation of *y*_*i*_ given the other parameters and data. We also let $Q(\alpha,\beta,\mu,\sigma ^{2},\rho) \equiv \mathbb {E}(\ell (\mathbf {x},\alpha,\beta,\mu,\sigma ^{2},\rho)|\text {rest})$ be the expectation of the log-likelihood given the other parameters and data. In particular

$$\begin{aligned} &Q(\alpha,\beta,\mu,\sigma^{2},\rho) \\ &= \sum\limits_{i=1}^{n} \left[(1-z_{i})\left(\alpha \log(\beta) - \log \Gamma(\alpha)\right.\right.\\ &\left.\quad+ (\alpha - 1) \log(x_{i}) - \beta x_{i} \right)\\ &\quad+ z_{i} \left(- \frac{1}{2} \log \left(2 \pi \sigma^{2}\right) - \frac{(x_{i}-\mu)^{2}}{2\sigma^{2}} \right) \\ &\left.\quad+ z_{i} \log \rho + (1-z_{i})\log(1-\rho) \right]. \end{aligned} $$

and $z_{i} = 1/(1 + e^{-\eta _{i}})\phantom {\dot {i}\!}$, for *i*=1,2,…,*n* where *η*_*i*_ is given by

$$\begin{array}{l} \eta_{i} = \widehat{\alpha} \log \left(\widehat{\beta}\right) - \log\Gamma \left(\widehat{\alpha}\right) + \left(\widehat{\alpha}-1\right) \log(x_{i}) - \widehat{\beta}x_{i} \\ \quad \quad \quad + \frac{1}{2}\log \left(2\pi\widehat{\sigma}^{2}\right) + \frac{\left(x_{i} - \widehat{\mu}\right)^{2}}{2\widehat{\sigma}^{2}} + \log\left(\frac{\widehat{\rho}}{1 - \widehat{\rho}}\right). \end{array} $$

The above describes the expectation step, while the following parameter updates describe the M-step,

$$\begin{array}{c} \widehat{\mu} = \frac{\sum z_{i} x_{i}}{\sum z_{i}}, \qquad \widehat{\sigma}^{2} = \frac{\sum z_{i} \left(x_{i} - \widehat{\mu}\right)^{2}}{\sum z_{i}}, \\ [2ex] \widehat{\alpha} \,=\, \text{igamma} \left(\frac{\sum \left(\log \widehat{\beta} + \log x_{i}\right)\left(1-z_{i}\right)}{\sum(1-z_{i})}\right), \qquad \widehat{\beta} \,=\, \frac{\widehat{\alpha} \sum (1-z_{i})}{\sum x_{i}(1-z_{i})}, \\ [2ex] \text{and} \qquad \widehat{\rho} = \frac{\sum z_{i}}{n}. \end{array} $$

where igamma is the inverse gamma function, implemented in R within the package *d* *i* *s* *t* *r*. The EM updates, as indicated by hat symbol, are made until there is negligible change in the parameter updates, or until a maximum number of iterations is reached. Initial values of *z*_*i*_,*i*=1,2,...,*n* are made by randomly generating from *n* independent *B*(1,0.5) distributions. After the algorithm converges, cell *i* is called “highly expressed” if *z*_*i*_≥0.5 and “lowly expressed” otherwise.

This mixture modeling framework was applied to each single cell RNA-Seq dataset separately. The result is a ternary matrix, containing values 0 (no expression), 1 (low expression) and 2 (high expression), and NA (missing values) with the same number of rows and columns as the log2CPM matrix. For each dataset, cell *i* and gene *j* the entries of the ternary matrix is

$$\begin{aligned} a_{ij} = \left \{\begin{array}{lc} 0, & \text{if}\ y_{ij}=0\\ 1, & \text{if cell \textit{i} classified to gamma component for gene \textit{j}}\\ 2, & \text{if cell \textit{i} classified to normal component for gene \textit{j}}\\ \text{NA}, & \text{if}\ y_{ij}\!>\!0 \text{\quad\!\! but not enough cells to fit model for gene \textit{j}} \end{array}\right. \end{aligned} $$

### Contextualizing genes to improve mixture modeling

We considered that there would be a large number of genes for which only a few cells have non-zero log2CPM values, rendering accurate fitting of the gamma-normal mixture models difficult. To ameliorate this issue we incorporated log2CPM values of ten other randomly selected genes and performed the EM algorithm. This was repeated ten times for each gene, and the majority ternary value of the ten repetitions taken as the final ternary value. Ties were dealt with in a conservative manner, that is, that the smaller value was chosen as the final ternary value for that gene and cell in the case of a tie.

### Curating an olfactory gene list

In order to further interrogate the data for biological relevance, we curated a set of genes of interest using Gene Ontology (GO) using the R packages GO.db v3.2.2 and org.Mm.eg.db v3.2.3. GO terms were queried using the search term “olfa”, resulting in a set of 33 terms related to olfactory processes such as ‘olfactory receptor activity’, and a set of 1129 genes that belong to these GO terms.

### Identifying transcriptionally active and coactive genes

We supposed that genes with a higher level of expression in given cells are in an active state, and thus warranted further examination. We determined that genes were ‘active’ in cells if they were classified into the normal component of the mixture model. We also wanted to characterize which genes tended to be in this ‘active’ state together for cells, i.e. coactive. In particular we generated a coactivation matrix given by *b*_*i*{*jk*}_=1{*a*_*ij*_=2,*a*_*ik*_=2} for *i*=1,2,…*n*_*g*_,*j*=1,2,…,*n*_*g*_, and *k*=1,2,…,*n*,*n*_*g*_ the number of genes and *n* the number of cells. Following this we could aim to identify what coactive pairs of genes were common with known markers of cell types.

### Identifying coactivation with known maturity markers

Next we aimed to understand which genes are markers for maturity of olfactory sensory neurons. A number of transcriptional markers are known for cell maturity and immaturity, such as *OMP* and *GAP43*, respectively [4, 18–21]. Using our estimates of transcriptionally active genes and cells, we considered coactivation of genes with these markers. We restricted cells to those that were active for *OMP* and not for *GAP43* as mature cells, and those active for *GAP43* and not for *OMP* as immature cells, and tested for coactivation among all genes in the transcriptome via Fisher’s exact test. Genes with Bonferroni-corrected *P*-values below 0.01 were considered as significantly coactivated with either *OMP* or *GAP43*.

By way of evaluation of these identified marker genes, we curated lists of genes that have previous evidence as markers for mature or immature OSNs. We used a set of 8 mature-specific and 10 immature-specific marker genes from Tan et al. [4] and a set of 691 mature-specific and 847 immature-specific marker genes from Nickell et al. [22], resulting in a combined list of 692 mature-specific and 851 immature-specific marker gene names, taking into account that multiple gene name aliases may exist. Also we note that this list of identified marker genes is not exhaustive and there may be other genes that are not captured in this curated list.

### Weighting coactive gene networks per cell by uniqueness

Next we attempted to better understand the variation of combinations of pairs of genes simultaneously expressed among the cells. In particular we wanted to study what gene pairs were uniquely coactive among the cells, distinguishing it from the overall population of cells. We did this by initially building gene-gene networks for each cell, taking the fully connected network of coactive genes. The number of nodes in this network is equal to the number of active genes for that cell *N*, and the number of edges is ${N \choose 2}$. In order to extract biologically meaningful characteristics we next incorporated a weighting per edge that took into account how often the edge was observed among the entire set of cell networks. An edge was removed if it prevalent, that is, if it was observed in more than 1% of the population of cells, resulting in a network of edges that were more uniquely coactive in that cell compared to the cell population. To ensure the robustness of the network characteristics observed, we also perturbed the threshold for prevalent edges, testing for 0.5, 1, 2, 3, 4, and 5%.

## Results

### Gamma-normal mixture is versatile for a number of transcriptional profiles

**Fig. 1:**
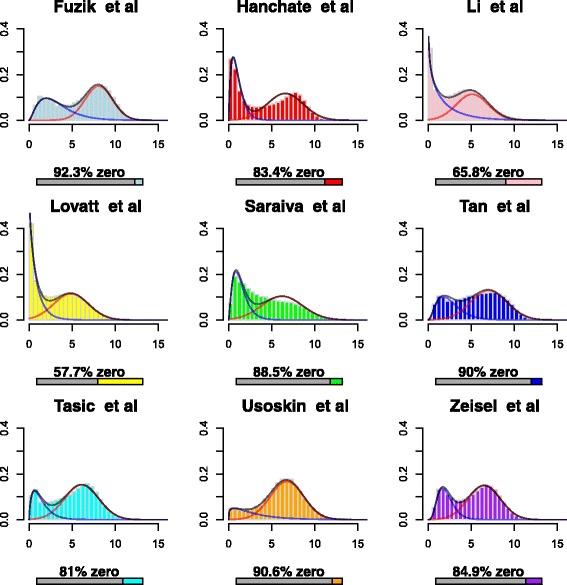
Histograms of log2CPM values of for all genes and cells within each dataset. *Zero* values are removed from the histograms, and the percentage of zero-values given for each dataset. *Black lines* represent the mixture model and the other two *blue* and *red colored lines* represent the gamma and normal mixture components respectively

We found that using a gamma-normal mixture model was suitable for accommodating the different empirical densities of the neuronal scRNA-Seq data. Figure 1 shows histograms of log2CPM values for all genes and cells for each dataset, with zeros removed. We found that while some datasets tended to have lower percentage of zeros (e.g. Li et al. [23] and Lovatt et al. [24]) resulting in a peak close to zero, the gamma-normal model was able to fit even this aspect of the data well.

**Fig. 2:**
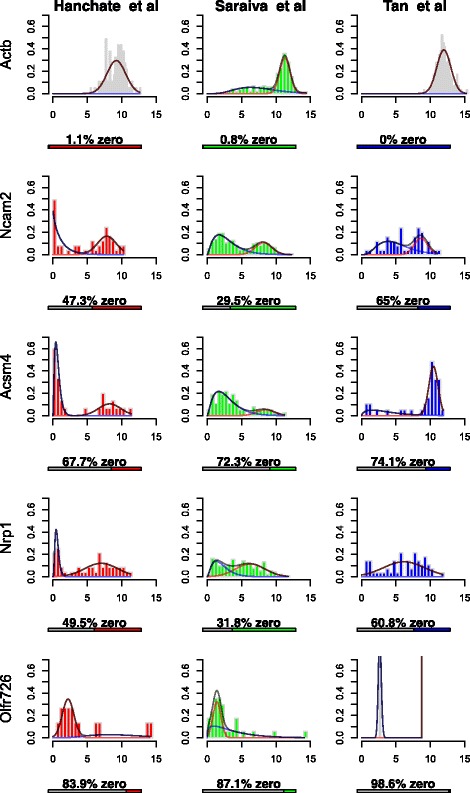
Histograms of log2CPM values of cells for particular genes (*ACTB, NCAM2, ACSM4, NRP1, OLFR726*) for three datasets Hanchate et al., Saraiva et al., and Tan et al. *Black lines* represent the mixture model and the other two *blue* and *red colored lines* represent the gamma and normal mixture components respectively. Performance of the mixture modeling framework can break down with few non-zero cells

However, since genes can have different dynamic ranges due to various technical effects (e.g. amplification or GC content bias), it is more suitable to estimate parameters of the gamma-normal mixture on a per-gene basis. Figure 2 shows histograms of log2CPM values for genes *ACTB, NCAM2, ACSM4, NRP1, OLFR726*, for datasets Hanchate et al. [25], Saraiva et al. [15] and Tan et al. [4], as well as the estimated gamma-normal mixture model densities. These three datasets were chosen as they all profile olfactory sensory neurons (OSNs), allowing for more direct comparisons without having to account for specific cell-type differences. The modeling framework identifies when the gene is highly expressed for all cells (*ACTB* a known housekeeping gene), as well as reasonable estimates for mixtures of lowly and highly expressed genes. However when there are too few cells with non-zero log2CPM values then the modeling framework can break down, for example the gene *OLFR726* for Tan et al. [4] there are only 2 cells with non-zero log2CPM values. We found that contextualizing genes enabled for these cells to be classified more accurately by including more data points into the mixture model. Contextualizing genes resulted in removal of missing values due to too few data points and further increased the difference between log2CPM values for genes and cells classified as 1 (lowly expressed) and 2 (highly expressed) (Additional file 1).

### Incorporating ternary data slightly improves read depth effects within datasets and facilitates clustering of cells

**Fig. 3:**
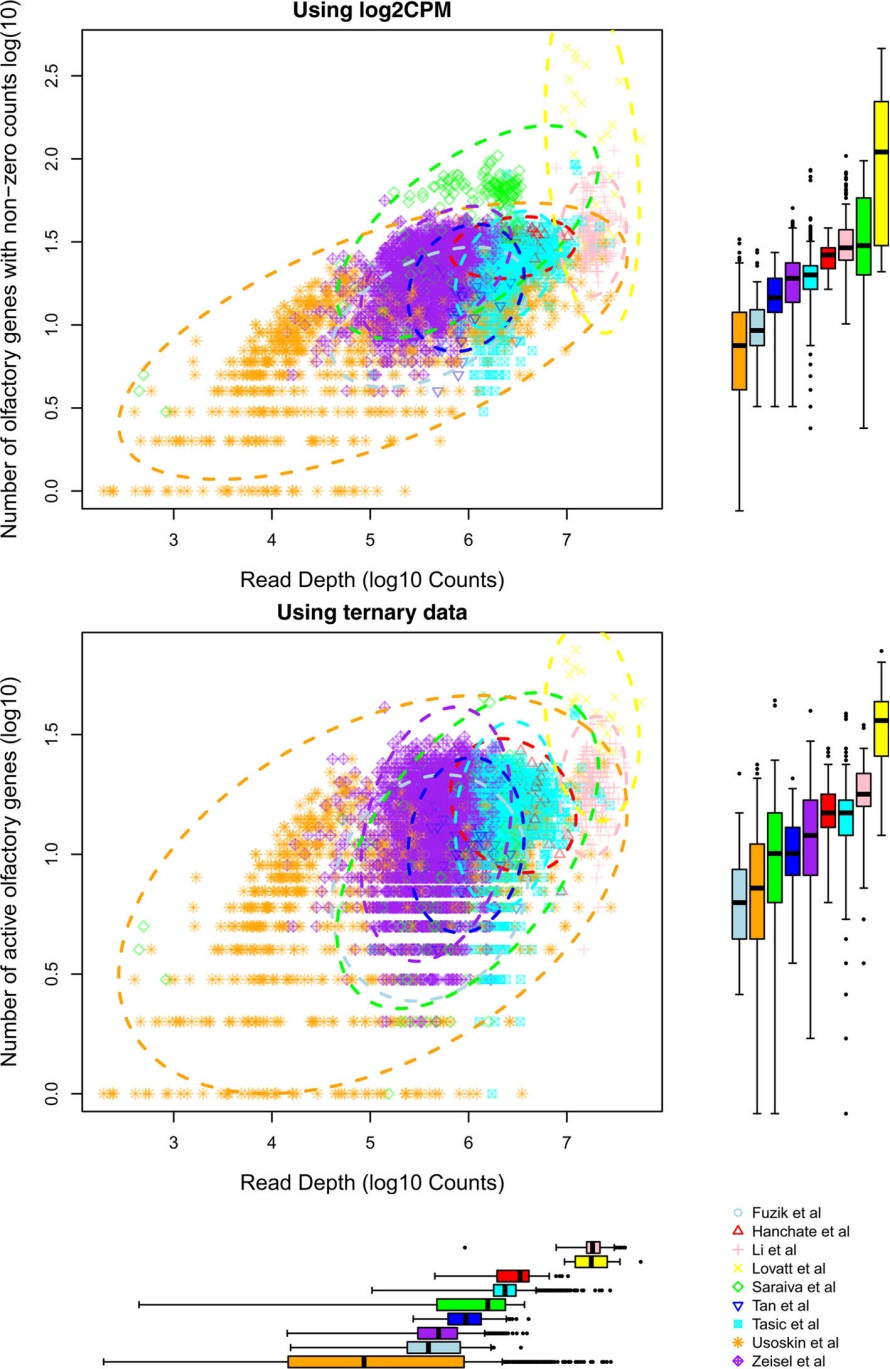
Scatterplots of total read depth versus number of non-zero log2CPM values (*top left*) and (*middle left*) number of active genes using genes related to olfactory system. Boxplots (*top right*, *middle right*, respectively) are of the number of non-zero log2CPM values and number of active genes using genes related to the olfactory system respectively, split by dataset. The last boxplot (*bottom left*) is of total read depth of cells from various datasets. We observe some relationship between total read depth and number of non-zero genes (*top left*), which is slightly diminished when comparing total read depth to the number of active genes (*middle left*) for datasets with lower total read depth

Next we considered what impact the total depth of sequencing had on the detection of genes. We found that in general as read depth tends to increase, the number of non-zero count genes also tends to increase (Additional file 2), however it seems that this effect is strongest when read depth is relatively low. This is important since different datasets (e.g. Usoskin et al.) have a very large dynamic range along the total read depth of the cells, and thus the number of identified genes would be biased. This also hints towards how deeply one should sequence the mRNA within a cell to be confident of capturing enough read counts for the data to be of further use in the analysis. We found after generating ternary matrices by fitting gene-wise gamma-normal mixture models, and considering the set of genes related to olfactory GO terms that this observed relationship between read depth and number of highly expressed genes was slightly diminished (Fig. 3). However the effect of read depth and number of active genes persists for some datasets, most notably that related to Usoskin et al. Additional file 3 displays the number of non-zero count genes against number of active genes, showing that the largest change occurs with data from Lovatt et al., indicated by the fitted line.

**Fig. 4:**
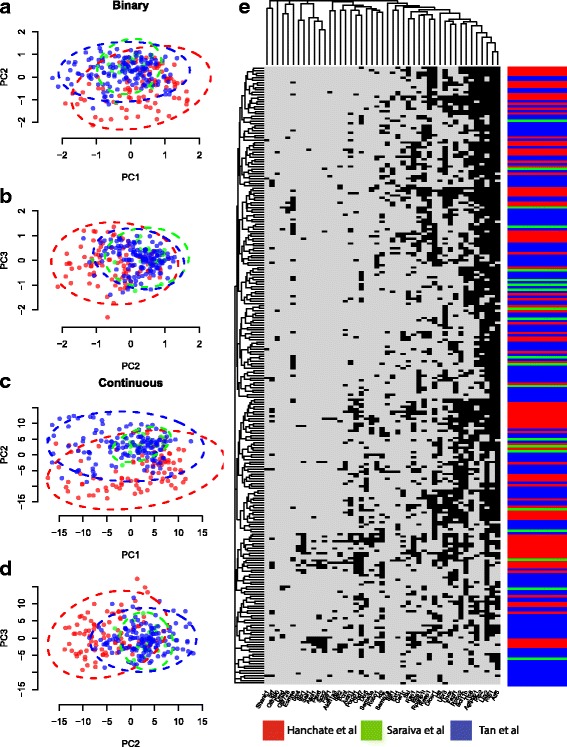
Principal components scores (PC1 vs PC2 and PC2 vs PC3) for binary values (panels **a** and **b**) and continuous log2CPM values (panels **c** and **d**) for cells from Hanchate et al. (*red*), Saraiva et al. (*green*) and Tan et al. (*blue*). Heatmap (panel **e**) of olfactory system genes and cells from Hanchate et al. (*red bars*), Saraiva et al. (*green bars*) and Tan et al. (*blue bars*), using binary values (*black* for ‘active’ and *light gray* for ‘inactive’). Only cells from Saraiva et al. dataset that passed the quality control [15] were included here

From this point on we focused on the olfactory sensory neuron datasets Hanchate et al., Saraiva et al. and Tan et al., and on genes related to the olfactory system as curated from GO, as this allowed us to combine and analyze data sets within the context of consistent cell types. We removed cells from the Saraiva et al. dataset that were removed in the original analysis, due to various technical effects such as cell clumping or breakage of cells [15]. Our interest lies in only active genes, so we converted the ternary matrices to binary matrices by setting values of 0 or 1 as 0, and values of 2 as 1. Thus the binary matrix represented 0 for no or low expression state, and 1 for a high or active expression state. In order to ensure that this data transformation led to increased comparability, or effective standardization, of the three transformed datasets, we compared the binary matrix to the corresponding matrix of log2CPM values in terms of classification performance. Figure 4 (left) shows the principal components analysis (PCA) for both the binary and continuous data, and we observe greater overlap of cells among the binary data than the continuous data. The hierarchical clustered heatmap of binary values in Fig. 4 (right) shows the cells, colored by dataset, are well mixed between datasets. In order to quantify what we observe in the figures, we considered how cells can be attributed to their original dataset via k-nearest neighbors classification. Since these cells belong to the same cell-type, we can assume that differences between these cells arise from non-biological factors such as technical differences. Thus if we observe a diminished classification accuracy of a cell to its original dataset label, then we can conclude that the transformation of the data results in increased comparability of the cells across the individual datasets. Indeed, performing a k-nearest neighbors classification on the originating dataset, the leave-one-out cross validation accuracy is diminished for the binary data, 66.7%, than the continuous, 71.4%, further assuring us that dataset specific effects are largely removed by transforming the data into binary active/non-active states.

### Investigating coactivation with known maturity markers recovers other known markers and integrating datasets reveals new putative markers for cell maturity

We tested for coactivation, that is, simultaneous active states, of genes between mature and immature cells in three cases involving OSN datasets. We defined immature OSNs as those cells that were active for the gene *GAP43* and inactive for *OMP*, and mature OSNs as those cells that were active for *OMP* and inactive for *GAP43*, as these genes have been previously described as transcriptional markers for cell maturity and immaturity respectively [18–21]. Cells that were either active for both *GAP43* and *OMP* or not active for either were not included for further testing of coactivation. We tested for coactivation of genes to these cell combinations using Fisher’s Exact Test, taking note of the gene in which coactivation occurred (coactivating with *OMP* suggests a mature marker or coactivating with *GAP43* suggests immature marker), thereby identifying if the tested gene was related positively toward maturity of immaturity. We applied this test in three cases: separately to the Hanchate et al. dataset and Tan et al. dataset, and to the concatenated dataset of Tan et al. and Hanchate et al. Note that we did not further consider the Saraiva et al. dataset as their experimental protocol selected for only mature neurons, that is those cells expressing *OMP*.

By way of evaluation, we compared the lists of putative markers for these three cases using a reference list of OSN mature and immature marker genes from Nickell et al. [22] and Tan et al. [4], a list of 692 mature genes, and 851 immature genes. This list of genes stems from literature-based and gene expression analysis and we note it may not be exhaustive, thus cannot be treated as a gold-standard positive set of genes. Comparison of our analyses of the two individual and merged datasets with the ‘reference’ gene list showed 95 of the 152 (62.5%) Tan et al., 27 of the 34 (79.4%) Hanchate et al., and 149 of the 245 (60.8%) merged marker genes appeared in the reference mature list, and 45 of the 73 (61.6%) Tan et al., 11 of the 27 (40.7%) Hanchate et al., and 63 of the 120 (52.5%) merged marker genes appeared in with the reference immature list.

Our analysis of the merged data sets identified 40 candidate genes that co-activated with *OMP* but were not found to coactivate with *OMP* when the individual datasets were analyzed alone (Table 2 and Additional file 4). Of these 40 genes, three (*RTP1*, *RTP2*, *PDLIM1*) are expressed in mature OSNs [26, 27]. *RTP1* and *RTP2* encode for proteins that facilitate the transport of odorant receptors to the membrane surface, a critical component for functional maturation of OSNs. The function of *PDLIM1* in mature OSNs is unknown.

**Table 2:** Candidate mature markers with known olfactory/neuronal expression and/or function

Symbol	Name	Category	Function	Citation
Rtp1	Receptor transporter 1	Olfactory Related	Transports olfactory receptors to cell surface	[26]
Rtp2	Receptor transporter 2	Olfactory Related	Transports olfactory receptors to cell surface	[26]
Pdlim1	PDZ and LIM domain 1	Olfactory Related	Differential zonal expression in the olfactory epithelium	[27]
Nxph3	Neurexophilin 3	Olfactory Related	Activated by cAMP in OSNs	[28]
Ccdc114	Coiled-coil domain containing 114	Olfactory Related	Ciliogenesis	
Napa	N-ethylmaleimide sensitive fusion protein attachment protein alpha	Neural Processes	Regulates SNARE complex	[41]
Cacna1h	Calcium voltage gated subunit alpha 1H	Neural Processes	Ca2+ voltage gated ion channel	[42]
Car2	Carbonic anhydrase 2	Neural Processes	Regulates neural excitation	[43, 44]
Arhgef28	Rho guanine nucleotide exchange factor 28	Neural Processes	Regulates axon growth and morphogenesis	[45]
Boc	Biregional cell adhesion molecule-related/down-regulated by oncogenes (Cdon) binding protein	Neural Processes	Specifies neural circuits in cortex and axon guidance candidate for commissural axon growth	[46, 47]
Sdc3	Syndecan 3	Neural Processes	Influences neurite outgrowth and cell spreading	[48, 49]
Tpm3	Tropomyosin 3, gamma	Neural Processes	Regulates neural polarity, and morphogenesis	[50, 51]
Nfatc1	Nuclear factor of activated T cells, cytoplasmic, calcineurin dependent 1	Neural Processes	Regulates calcium signaling	[52]
Ctsb	Cathepsin B	Neural Processes	Important for maturation and integrity of post natal CNS neurons	[53]
Cend1	Cell cycle exit and neuronal differentiation 1	Neural Processes	Marks the termination of neuron-generating divisions	[54]

*NXPH3* has been shown to be expressed in OSNs in a cAMP dependent manner [28]. Of the remaining 36 genes, none have been studied in the olfactory system. However, four are involved in ciliogenesis (*CCDC114*), synapse formation (*NAPA*), and excitation (*CACNA1H*, *CAR2*), consistent with a role in later stages of neuronal development. An additional seven have been shown to regulate axon guidance (*ARHGEF28*, *BOC*), neurite outgrowth (*SDC3*), neuronal morphology (*TPM3*), and differentiation (*NFATC1*, *CTSB*, *CEND1*). No clear association with neuronal specific function or expression could be easily inferred for the remaining 25 genes (Additional file 4), however, none are known markers for neural immaturity. Our findings support the utility of the merged mixed model approach for enhancing the detection of coactivated genes with merged scRNA-Seq data sets. Our approach identified 40 potential new markers, at least three of which are already known to be expressed in mature OSNs.

### Investigating coactivation of cells unravels network characteristics related to maturity of olfactory sensory neurons

**Fig. 5:**
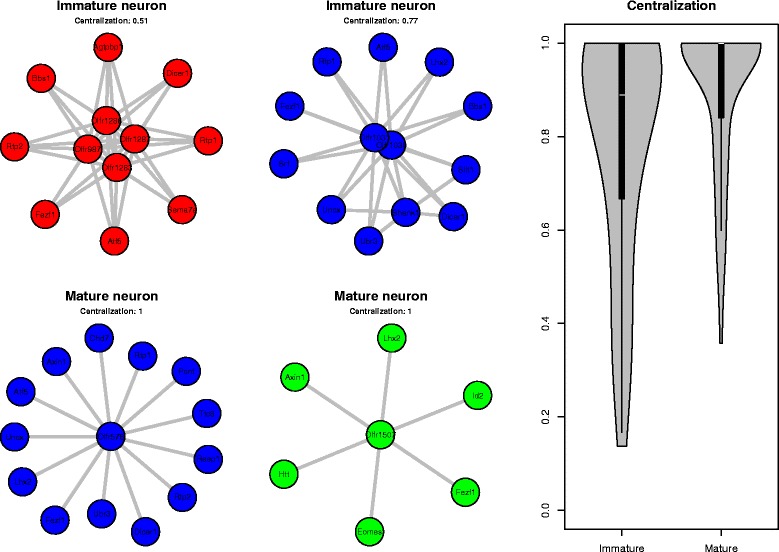
Examples of individual cell networks for immature neurons (*top row*) and mature neurons (*bottom row*). *Violin plot* shows centralization measures for immature and mature cells, with mature cell networks having a higher centralization than immature overall (*P*<0.02 two-sample t-test) Color indicates the dataset the cell originated from (*red* - Hanchate et al., *green* - Saraiva et al., and *blue* - Tan et al.). *Violin plot* of centralization scores for immature and mature neurons

We generated cell specific coactivation networks, by weighting edges on how unusually they appear in the dataset. Specifically, we included edges between two coactive genes if they appeared in less than 1% of the cell population, effectively weighting towards coactivation events that are rarely present than prevalent coactivating events. Upon examining some of these individual cell networks, it appeared that some had a very clear hub-partner topology, characterized by many partners leading to one or two nodes and no other connections (Fig. 5 bottom row), and others were more dense in the number of connections between different nodes (Fig. 5 top row). This suggests that for some cells, a single gene is uniquely activating, and thus coactivation occurs with the other genes that may be active in more cells, whereas for others there are a number of genes appearing uniquely, lending itself to a more densely connected network. In order to identify possible reasons for these different topologies, we considered comparing the centralization measures between cells that are mature OSNs and immature OSNs. Centralization is a measure of how central connections are towards some nodes, and are higher in networks with hub-partner topology [29]. As described earlier, we identified immature OSNs as those cells that were active for the gene *GAP43* and inactive for *OMP*, and mature OSNs as those cells that were active for *OMP* and inactive for *GAP43*. We considered only non-trivial networks with at least 5 nodes, resulting in 111 individual mature cell networks and 39 individual immature cell networks. We found that mature cell networks tend to be more central than immature cell networks (*P*<0.01, two-sided two-sample t-test). To ensure robustness of this result to choice of thresholds, we also compared networks with only edges appearing in less than 0.5, 1, 2, 3, 4, and 5% of the cell population. In all cases we observed a significant difference in centralization between the two groups (*P*-values 0.012, 0.013, 0.0032, 0.003, 0.0011, and 0.00046 respectively, two-sided two-sample t-test). Some representative cells from these groups are shown in Fig. 5. The entire set of non-trivial networks is shown in Additional file 1.

## Discussion and conclusions

In this paper we propose a method to identify transcriptionally active (highly expressed) gene patterns in single cell RNA-Seq data. This was achieved by employing a gamma-normal mixture modeling approach. This gene expression classification further enabled key observations in neuronal cell quality control, and facilitated examination of maturity markers with improved identification in combining datasets.

There has been some discussion as to what causes the apparent bimodal distribution of scRNA-Seq data, including attributing these highly expressed genes to transcriptional bursting [14], referring to very rapid production of RNA occurring in bursts, owing to the stochastic nature of transcription in the cell. Indeed, transcriptional bursting has been explored both theoretically [30], within cell-line studies [31], and in the context of scRNA-Seq data [13]. Our mixture modeling framework enables identification of genes for which the cell is possibly undergoing transcriptional bursting or is highly expressed, as those that were deemed ‘active’ throughout this paper, and thus potentially can be used to analyze bursting states given a suitable experimental protocol.

Potential limitations of the method introduced in this paper is the treatment of zero counts. In the case where there are many false positive reads, that is, reads mapped to a gene when in fact there is no underlying transcription occurring, the error may propagate and cells be classified as lowly or less likely highly expressed. A strategy for dealing with this issue may be to incorporate a third component into the gamma-normal mixture model, where the third component has a very high probability density at zero, but also incorporates a non-zero probability for non-zero values. Of course, this requires that the proportion of non-zero values can be estimated somehow. However, in this paper our key observations stemmed from focusing specifically on active genes, and potential issues associated with false positive reads are negligible in this setting.

Additional methodological developments are needed for datasets and genes that do not have a clear bimodal distribution of expression values. These are cells with very little to no highly active genes and did not have enough cells to accurately fit the gamma-normal mixture model, e.g. Fig. 2 for *OLFR726*. Given a suitable continuous normalization approach, this issue of not enough cells can be ameliorated by simply combining the cells into one large merged dataset. This of course is dependent on a reliable cross-dataset normalization strategy. Methods on batch correction [32] and normalization of bulk RNA-Seq [33–35] data exist, but it is not yet clear how applicable these approaches are given the unique characteristics of scRNA-Seq such as the abundance of zero values, with strides in effective normalization of scRNA-Seq data actively developing [36].

Using methods to identify active genes and coactive gene pairs within cells, we have been able to identify gene markers for olfactory sensory neuron maturity across multiple datasets, and to observe characteristics of cell-specific coactivation networks weighted by uniqueness. This unique way of exploring single cell RNA-Seq data has enabled interesting observations and future applications to other types of single cell RNA-Seq will be of interest.

## Additional files

Additional file 1
**Figure S1.** Violin plots of log2CPM values stratified by classification of 1 (lowly expressed), 2 (highly expressed) and NA (not enough data to classify) before (left of dashed line) and after (right of dashed line) employing contextualization of genes, resulting in better separation of log2CPM values between classes 1 and 2, and removal of missing values from the method. (PDF 50.5 KB)

Additional file 2
**Figure S2.** Scatterplots of total read depth versus number of non-zero log2CPM values (top left) and (middle left) number of active genes using all genes. Boxplots (top right, middle right, respectively) are of the number of non-zero log2CPM values and number of active genes using all genes respectively, split by dataset. The last boxplot (bottom left) is of total read depth of cells from various datasets. Unsurprisingly, we observe some relationship between total read depth and number of non-zero genes (top left), which is slightly diminished when comparing total read depth to the number of active genes (middle left) for datasets with lower total read depth. (PDF 455 KB)

Additional file 3
**Figure S3.** Scatterplot of number of olfactory genes with non-zero values against number of olfactory genes classified as active (highly expressed). The gray solid line is the diagonal line and other dotted lines are fitted lines for each dataset. (PDF 225 KB)

Additional file 4
**Table S1.** This xlsx file contains candidate mature markers with unknown olfactory/neuronal expression and/or function. (XLSX 8.15 KB)

Additional file 5 Cell-specific network graphs. This pdf file contains cell name (SRA or equivalent ID), visualization of the cell uniqueness network, its centralization score as well as its classification as a ‘mature’, ‘immature’ or ‘unsure’ of maturity cell, for all 211 non-trivial cell networks. (PDF 498 KB)

## Abbreviations

CPM: Counts per million
EM: Expectation maximization
ENA: European nucleotide archive
FISH: Fluorescence in situ hybridization
GEO: Gene expression omnibus
GO gene ontology; OSN: Olfactory sensory neuron
RPKM: Reads per kilobase per million
scRNA-Seq: Single cell RNA-Sequencing
SRA: Sequence read archive
