# Supplementary material for: Integrated single cell data analysis reveals cell specific networks and novel coactivation markers
Source: BMC Syst Biol. 2016 Dec 5;10(Suppl 5):127. doi: 10.1186/s12918-016-0370-4 (PMC5249008; doi:10.1186/s12918-016-0370-4)
Supplement: Additional file 1 — Figure S1. Violin plots of log2CPM values stratified by classification of 1 (lowly expressed), 2 (highly expressed) and NA (not enough data to classify) before (left of dashed line) and after (right of dashed line) employing contextualization of genes, resulting in better separation of log2CPM values between classes 1 and 2, and removal of missing values from the method. (PDF 50.5 KB) [file 12918_2016_370_MOESM1_ESM.pdf]

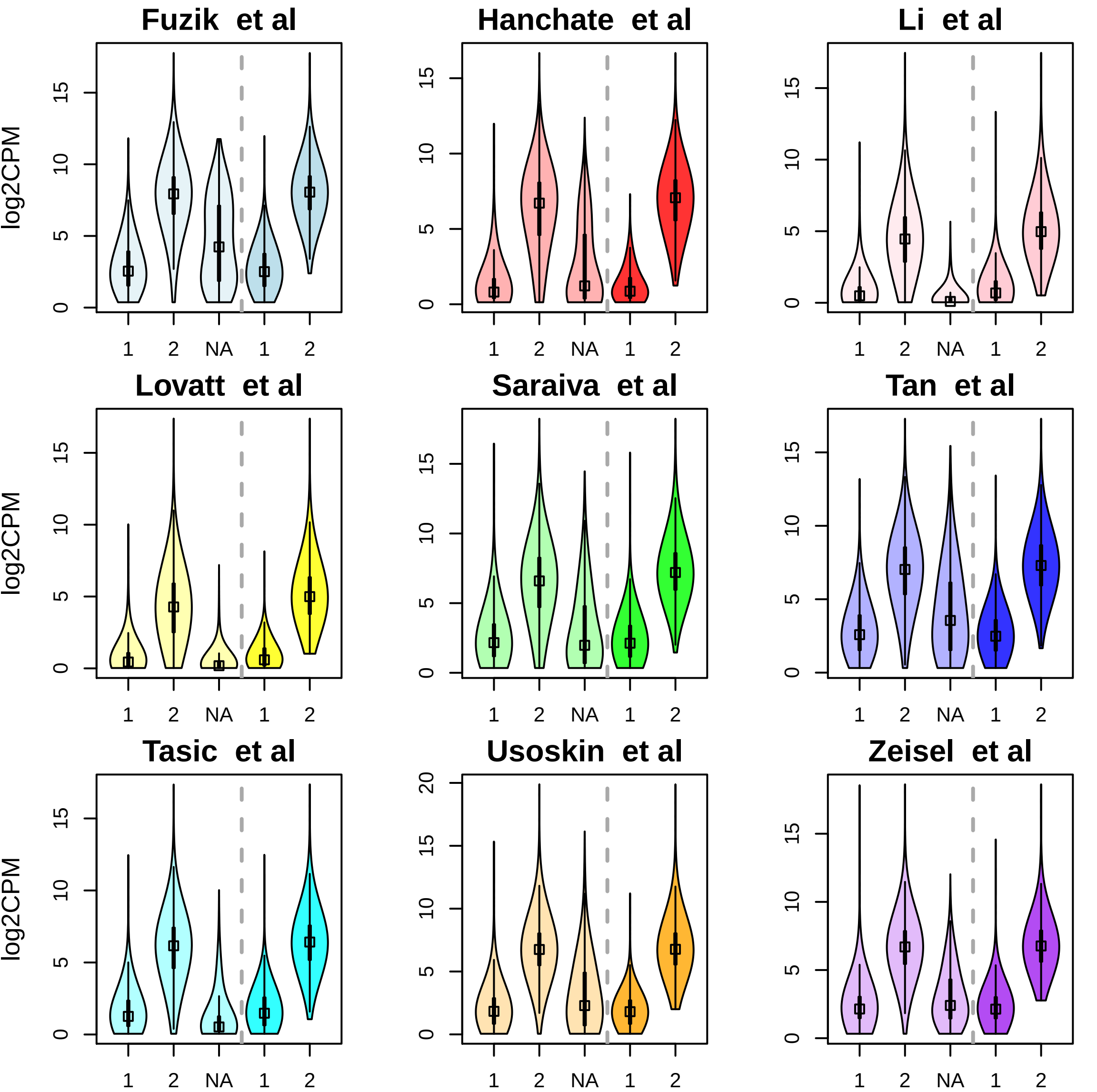
Fuzik et al
Hanchate et al
Li et al
Lovatt et al
Saraiva et al
Tan et al
Tasic et al
Usoskin et al
Zeisel et al
log2CPM
0
5
10
15
20
1
2
NA
