# Supplementary material for: Integrated single cell data analysis reveals cell specific networks and novel coactivation markers
Source: BMC Syst Biol. 2016 Dec 5;10(Suppl 5):127. doi: 10.1186/s12918-016-0370-4 (PMC5249008; doi:10.1186/s12918-016-0370-4)
Supplement: Additional file 2 — Figure S2. Scatterplots of total read depth versus number of non-zero log2CPM values (top left) and (middle left) number of active genes using all genes. Boxplots (top right, middle right, respectively) are of the number of non-zero log2CPM values and number of active genes using all genes respectively, split by dataset. The last boxplot (bottom left) is of total read depth of cells from various datasets. Unsurprisingly, we observe some relationship between total read depth and number of non-zero genes (top left), which is slightly diminished when comparing total read depth to the number of active genes (middle left) for datasets with lower total read depth. (PDF 455 KB) [file 12918_2016_370_MOESM2_ESM.pdf]

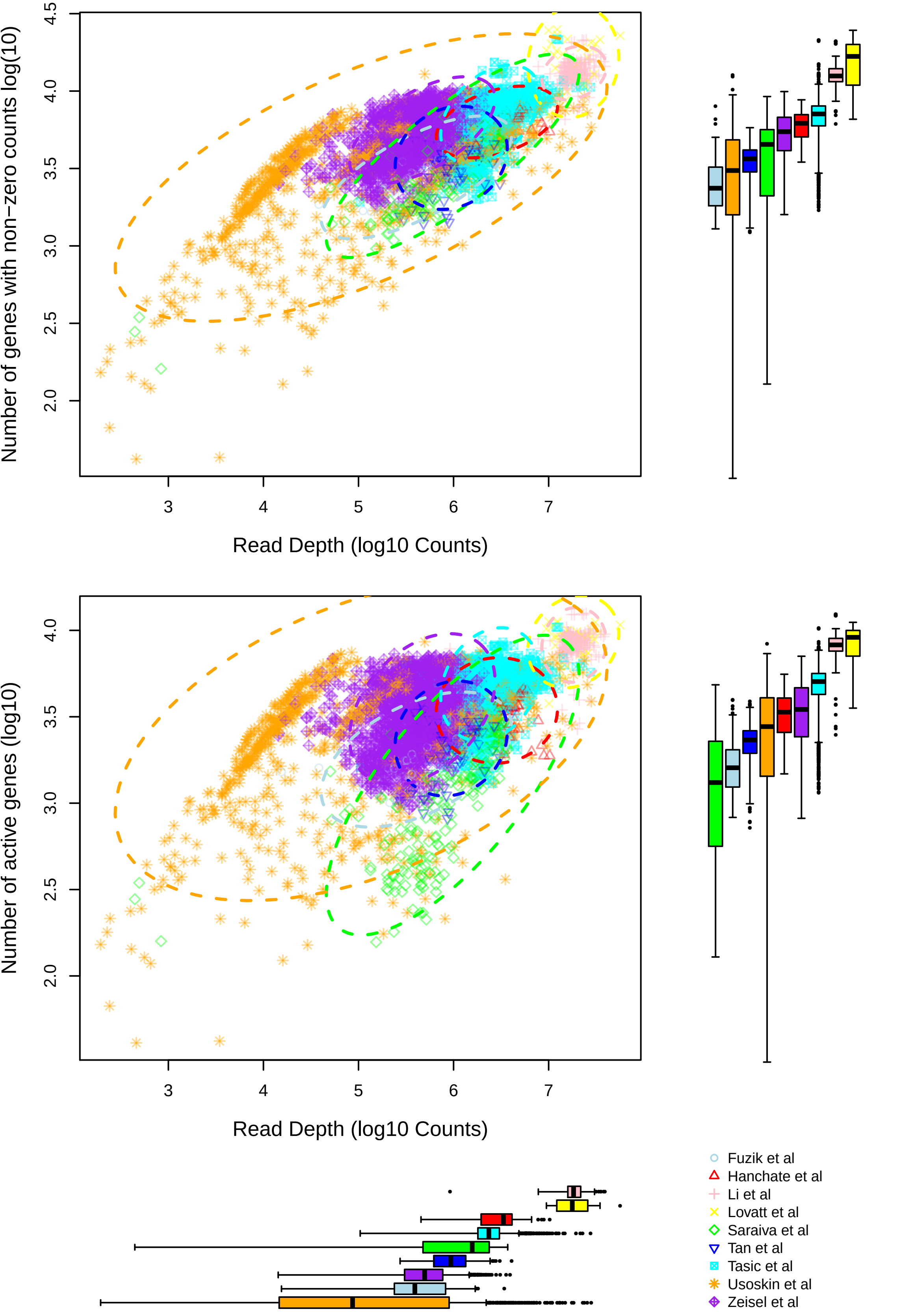

Number of genes with non−zero counts log(10)
4.5
4.0
3.5
3.0
2.5
2.0
3
4
5
6
7
Read Depth (log10 Counts)
Number of active genes (log10)
4.0
3.5
3.0
2.5
2.0
3
4
5
6
7
Read Depth (log10 Counts)
Fuzik et al
Hanchate et al
Li et al
Lovatt et al
Saraiva et al
Tan et al
Tasic et al
Usoskin et al
Zeisel et al
