# Supplementary figures and images for: Integrated single cell data analysis reveals cell specific networks and novel coactivation markers
Source: BMC Syst Biol. 2016 Dec 5;10(Suppl 5):127. doi: 10.1186/s12918-016-0370-4 (PMC5249008; doi:10.1186/s12918-016-0370-4)

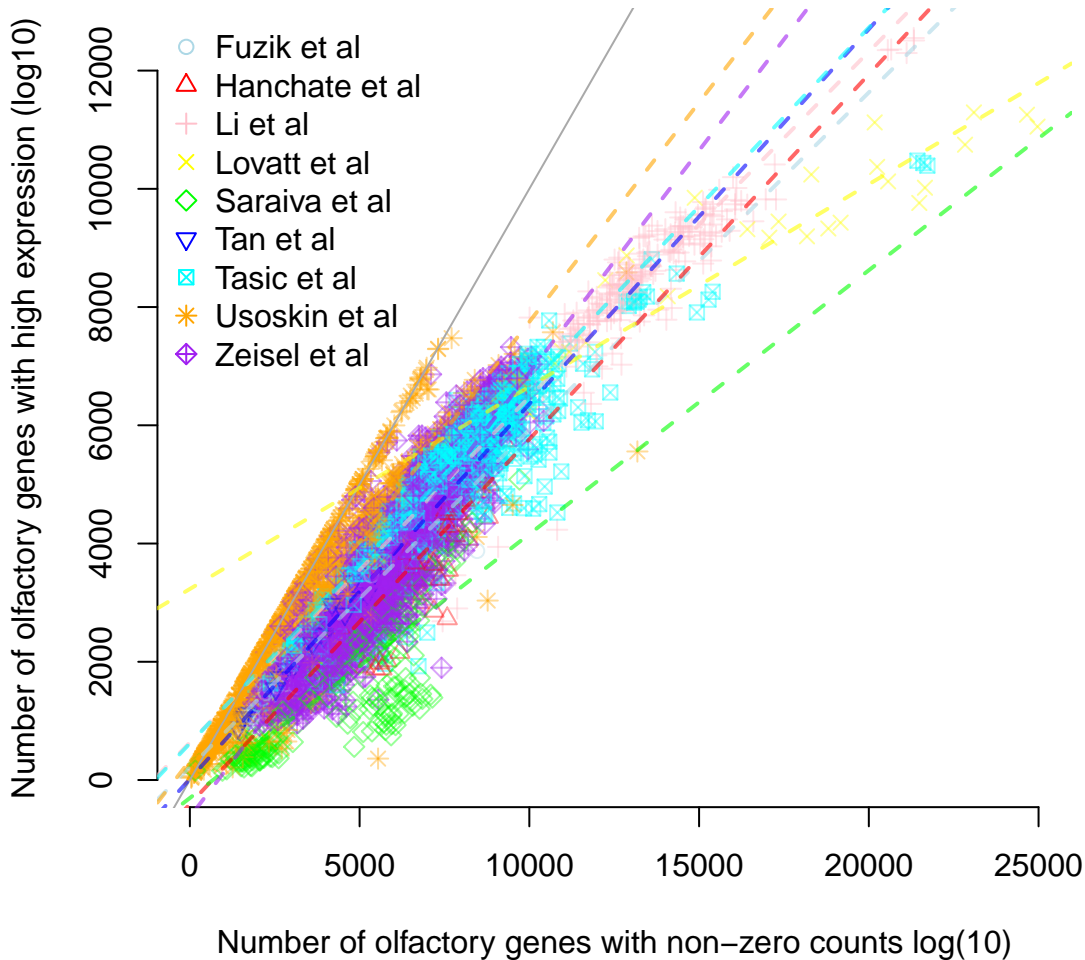

Supplement: Additional file 3 — Figure S3. Scatterplot of number of olfactory genes with non-zero values against number of olfactory genes classified as active (highly expressed). The gray solid line is the diagonal line and other dotted lines are fitted lines for each dataset. (PDF 225 KB) [file 12918_2016_370_MOESM3_ESM.pdf]
