# Supplementary material for: Integrated single cell data analysis reveals cell specific networks and novel coactivation markers
Source: BMC Syst Biol. 2016 Dec 5;10(Suppl 5):127. doi: 10.1186/s12918-016-0370-4 (PMC5249008; doi:10.1186/s12918-016-0370-4)
Supplement: Additional file 5 — Cell-specific network graphs. This pdf file contains cell name (SRA or equivalent ID), visualization of the cell uniqueness network, its centralization score as well as its classification as a ‘mature’, ‘immature’ or ‘unsure’ of maturity cell, for all 211 non-trivial cell networks. (PDF 498 KB) [file 12918_2016_370_MOESM5_ESM.pdf]

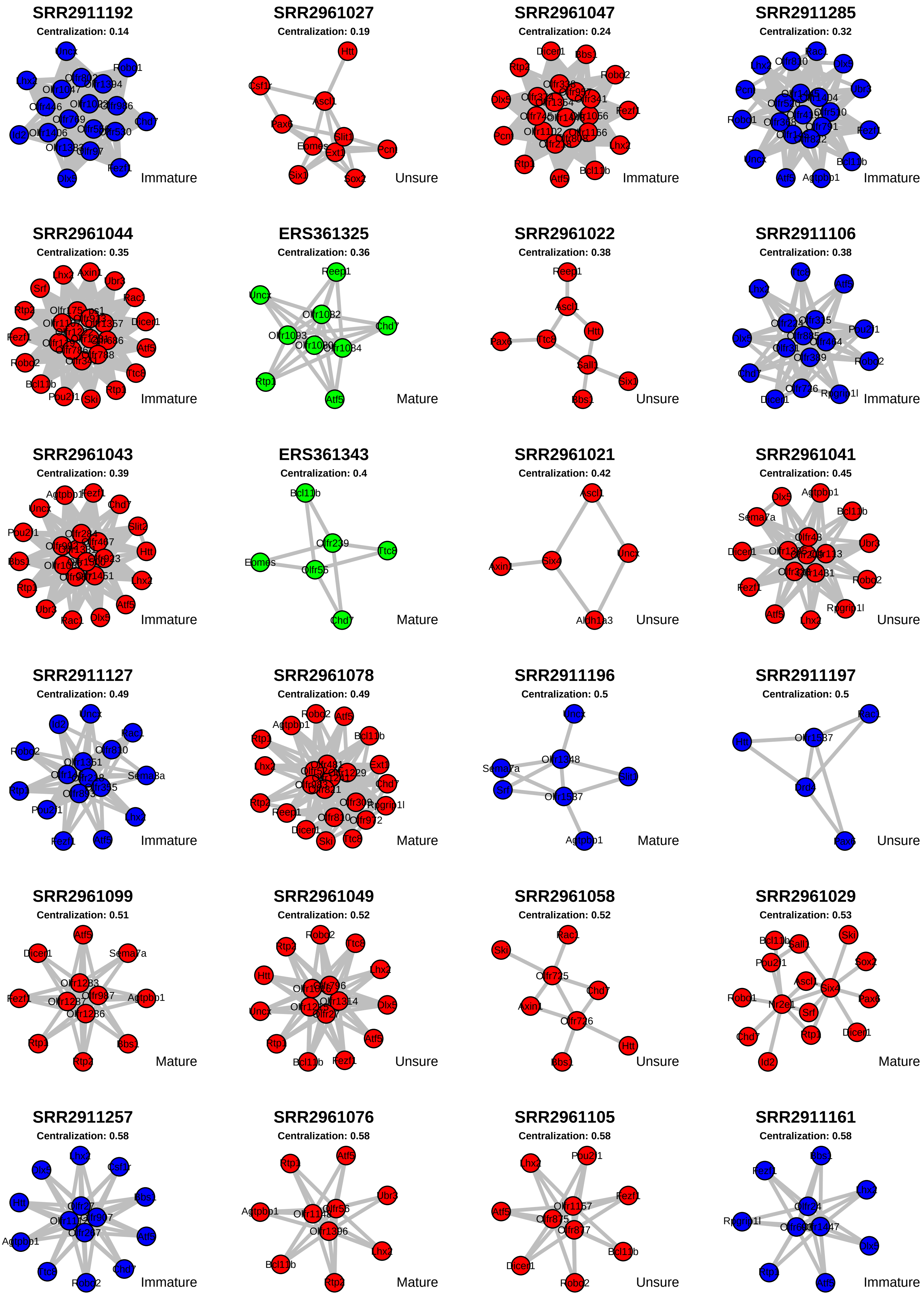
SRR2911192
Centralization: 0.14
Immature
SRR2961027
Centralization: 0.19
Unsure
SRR2961047
Centralization: 0.24
Immature
SRR2911285
Centralization: 0.32
Immature
SRR2961044
Centralization: 0.35
Immature
ERS361325
Centralization: 0.36
Mature
SRR2961022
Centralization: 0.38
Unsure
SRR2911106
Centralization: 0.38
Immature
SRR2961043
Centralization: 0.39
Immature
ERS361343
Centralization: 0.4
Mature
SRR2961021
Centralization: 0.42
Unsure
SRR2961041
Centralization: 0.45
Unsure
SRR2911127
Centralization: 0.49
Immature
SRR2961078
Centralization: 0.49
Mature
SRR2911196
Centralization: 0.5
Mature
SRR2911197
Centralization: 0.5
Unsure
SRR2961099
Centralization: 0.51
Mature
SRR2961049
Centralization: 0.52
Unsure
SRR2961058
Centralization: 0.52
Unsure
SRR2961029
Centralization: 0.53
Mature
SRR2911257
Centralization: 0.58
Immature
SRR2961076
Centralization: 0.58
Mature
SRR2961105
Centralization: 0.58
Unsure
SRR2911161
Centralization: 0.58
Immature

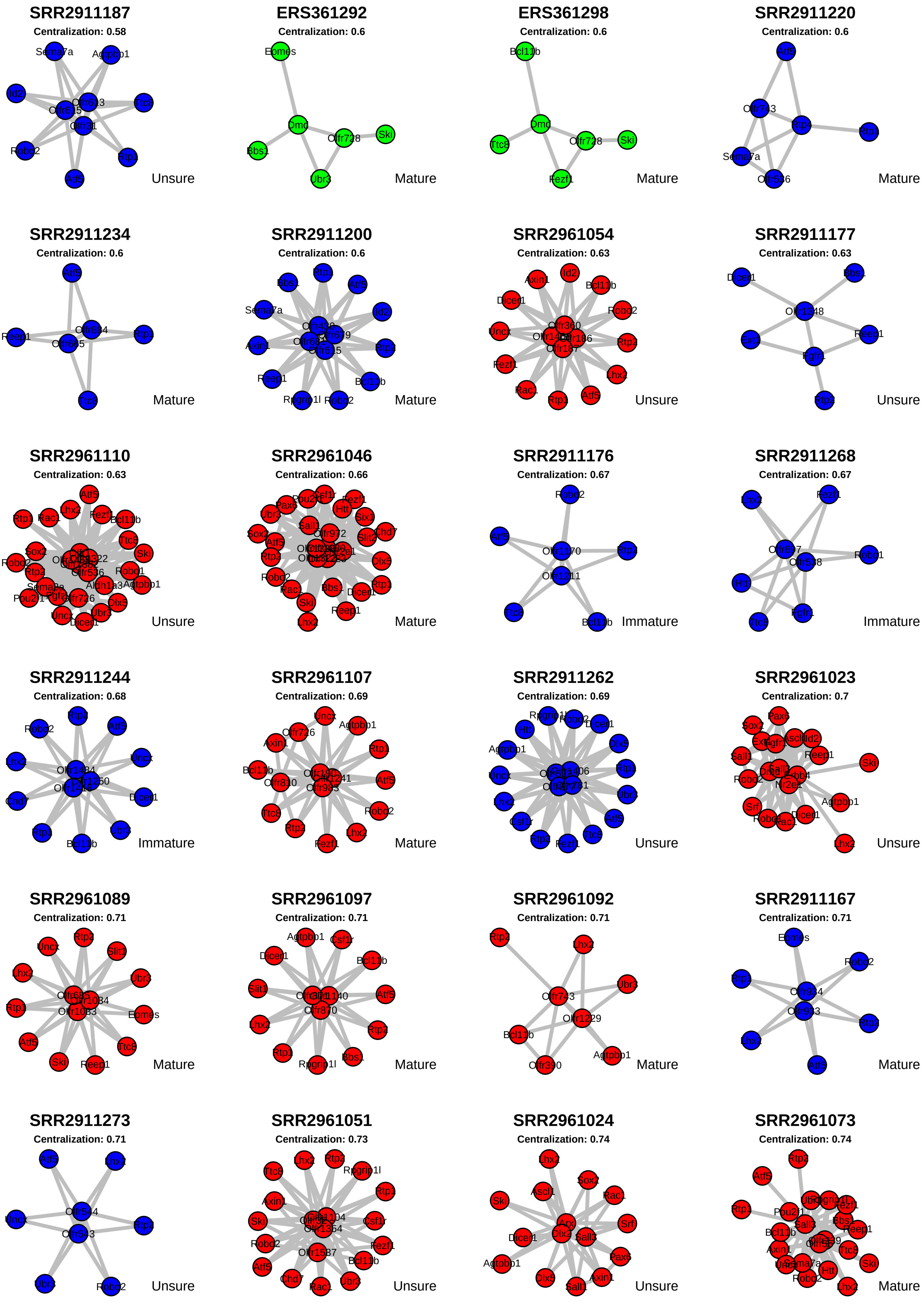
SRR2911187
Centralization: 0.58
Unsure
ERS361292
Centralization: 0.6
Mature
ERS361298
Centralization: 0.6
Mature
SRR2911220
Centralization: 0.6
Mature
SRR2911234
Centralization: 0.6
Mature
SRR2911200
Centralization: 0.6
Mature
SRR2961054
Centralization: 0.63
Unsure
SRR2911177
Centralization: 0.63
Unsure
SRR2961110
Centralization: 0.63
Unsure
SRR2961046
Centralization: 0.66
Mature
SRR2911176
Centralization: 0.67
Immature
SRR2911268
Centralization: 0.67
Immature
SRR2911244
Centralization: 0.68
Immature
SRR2961107
Centralization: 0.69
Mature
SRR2911262
Centralization: 0.69
Unsure
SRR2961023
Centralization: 0.7
Unsure
SRR2961089
Centralization: 0.71
Mature
SRR2961097
Centralization: 0.71
Mature
SRR2961092
Centralization: 0.71
Mature
SRR2911167
Centralization: 0.71
Mature
SRR2911273
Centralization: 0.71
Unsure
SRR2961051
Centralization: 0.73
Unsure
SRR2961024
Centralization: 0.74
Unsure
SRR2961073
Centralization: 0.74
Mature

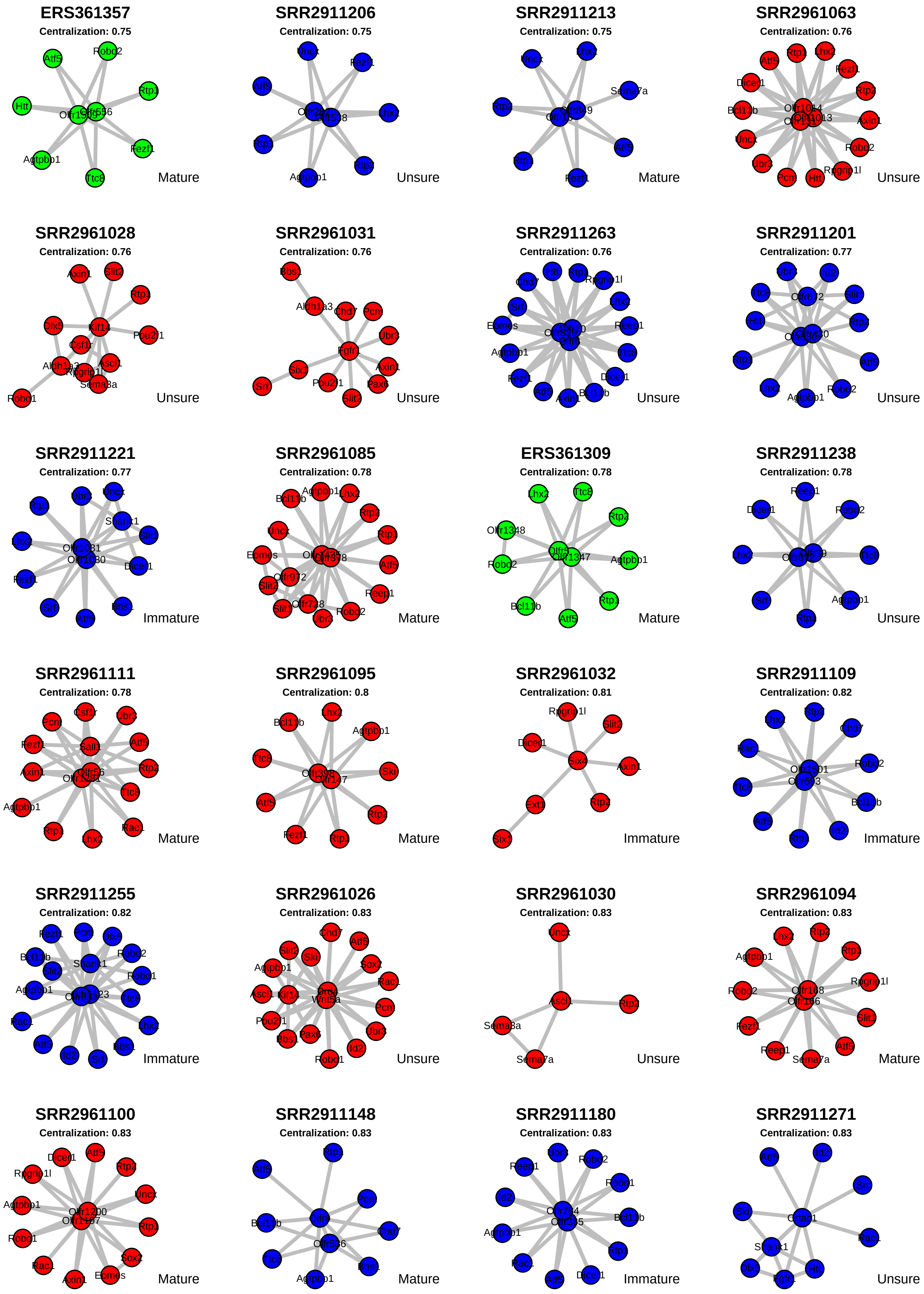
ERS361357
Centralization: 0.75
Mature
SRR2911206
Centralization: 0.75
Unsure
SRR2911213
Centralization: 0.75
Mature
SRR2961063
Centralization: 0.76
Unsure
SRR2961028
Centralization: 0.76
Unsure
SRR2961031
Centralization: 0.76
Unsure
SRR2911263
Centralization: 0.76
Unsure
SRR2911201
Centralization: 0.77
Unsure
SRR2911221
Centralization: 0.77
Immature
SRR2961085
Centralization: 0.78
Mature
ERS361309
Centralization: 0.78
Mature
SRR2911238
Centralization: 0.78
Unsure
SRR2961111
Centralization: 0.78
Mature
SRR2961095
Centralization: 0.8
Mature
SRR2961032
Centralization: 0.81
Immature
SRR2911109
Centralization: 0.82
Immature
SRR2911255
Centralization: 0.82
Immature
SRR2961026
Centralization: 0.83
Unsure
SRR2961030
Centralization: 0.83
Unsure
SRR2961094
Centralization: 0.83
Mature
SRR2961100
Centralization: 0.83
Mature
SRR2911148
Centralization: 0.83
Mature
SRR2911180
Centralization: 0.83
Immature
SRR2911271
Centralization: 0.83
Unsure

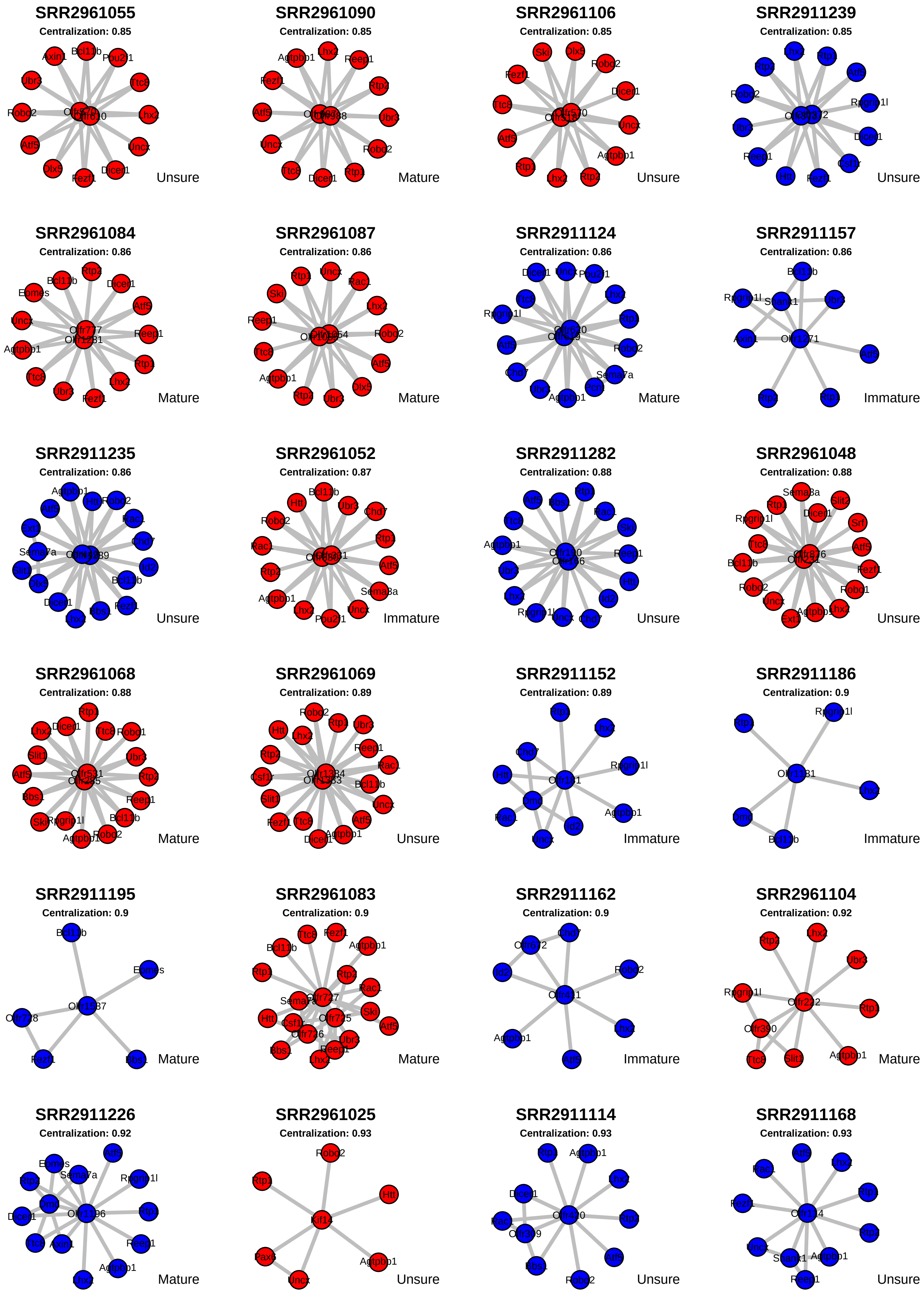
SRR2961055
Centralization: 0.85
Unsure
SRR2961090
Centralization: 0.85
Mature
SRR2961106
Centralization: 0.85
Unsure
SRR2911239
Centralization: 0.85
Unsure
SRR2961084
Centralization: 0.86
Mature
SRR2961087
Centralization: 0.86
Mature
SRR2911124
Centralization: 0.86
Mature
SRR2911157
Centralization: 0.86
Immature
SRR2911235
Centralization: 0.86
Unsure
SRR2961052
Centralization: 0.87
Immature
SRR2911282
Centralization: 0.88
Unsure
SRR2961048
Centralization: 0.88
Unsure
SRR2961068
Centralization: 0.88
Mature
SRR2961069
Centralization: 0.89
Unsure
SRR2911152
Centralization: 0.89
Immature
SRR2911186
Centralization: 0.9
Immature
SRR2911195
Centralization: 0.9
Mature
SRR2961083
Centralization: 0.9
Mature
SRR2911162
Centralization: 0.9
Immature
SRR2961104
Centralization: 0.92
Mature
SRR2911226
Centralization: 0.92
Mature
SRR2961025
Centralization: 0.93
Unsure
SRR2911114
Centralization: 0.93
Unsure
SRR2911168
Centralization: 0.93
Unsure

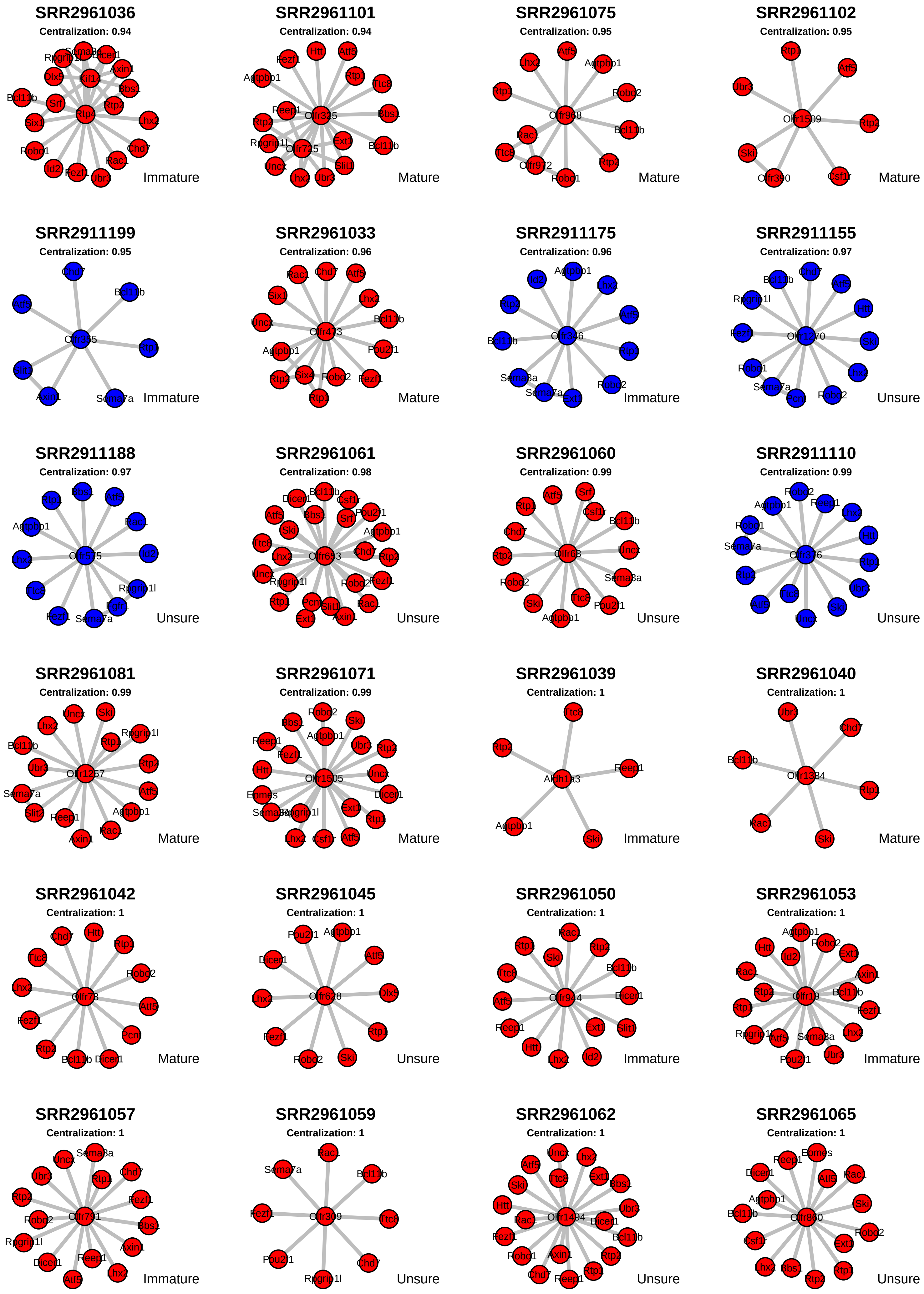

SRR2961036
Centralization: 0.94
Rtp4
Kif1a
Olx5
Srf
Axin1
Bbs1
Rtp2
Bcl11b
Six1
Lhx2
Robo1
Chd7
Id2
Fezf1
Ubr3
Rac1
Immature
SRR2961101
Centralization: 0.94
Olfr325
Htt
Atf5
Fezf1
Agtpbp1
Rtp1
Ttc8
Bbs1
Reep1
Rtp2
Rpgrip1l
Olfr725
Ext1
Bcl11b
Slit1
Unc
Lhx2
Ubr3
Mature
SRR2961075
Centralization: 0.95
Olfr968
Atf5
Lhx2
Agtpbp1
Rtp1
Robo2
Bcl11b
Rac1
Ttc8
Olfr972
Rtp2
Robo1
Mature
SRR2961102
Centralization: 0.95
Olfr1509
Rtp1
Atf5
Ubr3
Rtp2
Ski
Olfr390
Csf1r
Mature
SRR2911199
Centralization: 0.95
Olfr355
Chd7
Bcl11b
Atf5
Rtp2
Slit1
Axin1
Sema7a
Immature
SRR2961033
Centralization: 0.96
Olfr473
Rac1
Chd7
Atf5
Six1
Lhx2
Uncx
Bcl11b
Agtpbp1
Pou2f1
Rtp2
Six4
Robo2
Fezf1
Rtp1
Mature
SRR2911175
Centralization: 0.96
Olfr346
Id2
Agtpbp1
Lhx2
Rtp2
Atf5
Bcl11b
Rtp1
Sema3a
Sema7a
Ext1
Robo2
Immature
SRR2911155
Centralization: 0.97
Olfr1270
Bcl11b
Chd7
Atf5
Rpgrip1l
Htt
Fezf1
Ski
Robo1
Lhx2
Sema7a
Pcnt
Robo2
Unsure
SRR2911188
Centralization: 0.97
Olfr575
Rtp1
Bbs1
Atf5
Agtpbp1
Rac1
Lhx2
Id2
Ttc8
Rpgrip1l
Fgfr1
Fezf1
Sema7a
Unsure
SRR2961061
Centralization: 0.98
Olfr653
Dicer1
Bcl11b
Csf1r
Pou2f1
Atf5
Bbs1
Srf
Ski
Agtpbp1
Ttc8
Lhx2
Chd7
Rtp2
Uncx
Rpgrip1l
Robo2
Fezf1
Rtp1
Pcnt
Slit1
Rac1
Ext1
Axin1
Unsure
SRR2961060
Centralization: 0.99
Olfr68
Atf5
Srf
Rtp1
Csf1r
Bcl11b
Chd7
Rtp2
Uncx
Robo2
Sema3a
Ski
Ttc8
Pou2f1
Agtpbp1
Unsure
SRR2911110
Centralization: 0.99
Olfr376
Robo2
Agtpbp1
Reep1
Lhx2
Robo1
Htt
Sema7a
Rtp1
Rtp2
Ttc8
Ubr3
Atf5
Ski
Uncx
Unsure
SRR2961081
Centralization: 0.99
Olfr1257
Uncx
Ski
Lhx2
Rpgrip1l
Bcl11b
Rtp1
Ubr3
Rtp2
Sema7a
Atf5
Slit2
Reep1
Agtpbp1
Axin1
Rac1
Mature
SRR2961071
Centralization: 0.99
Olfr1505
Robo2
Bbs1
Ski
Agtpbp1
Reep1
Ubr3
Rtp2
Fezf1
Htt
Uncx
Eomes
Dicer1
Sema3a
Rpgrip1l
Ext1
Rtp1
Lhx2
Csf1r
Atf5
Mature
SRR2961039
Centralization: 1
Aldh1a3
Ttc8
Rtp2
Reep1
Agtpbp1
Ski
Immature
SRR2961040
Centralization: 1
Olfr1384
Ubr3
Chd7
Bcl11b
Rtp1
Rac1
Ski
Mature
SRR2961042
Centralization: 1
Olfr78
Chd7
Htt
Rtp1
Ttc8
Robo2
Lhx2
Atf5
Fezf1
Pcnt
Rtp2
Bcl11b
Dicer1
Mature
SRR2961045
Centralization: 1
Olfr628
Pou2f1
Agtpbp1
Dicer1
Atf5
Lhx2
Olx5
Rtp1
Fezf1
Robo2
Ski
Unsure
SRR2961050
Centralization: 1
Olfr944
Rac1
Rtp1
Rtp2
Ski
Bcl11b
Ttc8
Dicer1
Atf5
Reep1
Ext1
Slit1
Htt
Lhx2
Id2
Immature
SRR2961053
Centralization: 1
Olfr19
Agtpbp1
Htt
Robo2
Id2
Ext1
Rac1
Axin1
Rtp2
Bcl11b
Rtp1
Fezf1
Rpgrip1l
Atf5
Sema3a
Lhx2
Pou2f1
Ubr3
Immature
SRR2961057
Centralization: 1
Olfr791
Uncx
Sema3a
Ubr3
Rtp1
Chd7
Rtp2
Fezf1
Robo2
Bbs1
Rpgrip1l
Axin1
Reep1
Dicer1
Lhx2
Atf5
Immature
SRR2961059
Centralization: 1
Olfr309
Rac1
Sema7a
Bcl11b
Fezf1
Ttc8
Pou2f1
Chd7
Rpgrip1l
Unsure
SRR2961062
Centralization: 1
Olfr1494
Uncx
Lhx2
Atf5
Ttc8
Ext1
Ski
Bbs1
Htt
Ubr3
Rac1
Dicer1
Fezf1
Bcl11b
Robo1
Axin1
Rtp2
Chd7
Reep1
Rtp1
Unsure
SRR2961065
Centralization: 1
Olfr860
Eomes
Reep1
Dicer1
Atf5
Rac1
Agtpbp1
Bcl11b
Ski
Robo2
Csf1r
Ext1
Lhx2
Bbs1
Rtp1
Rtp2
Unsure

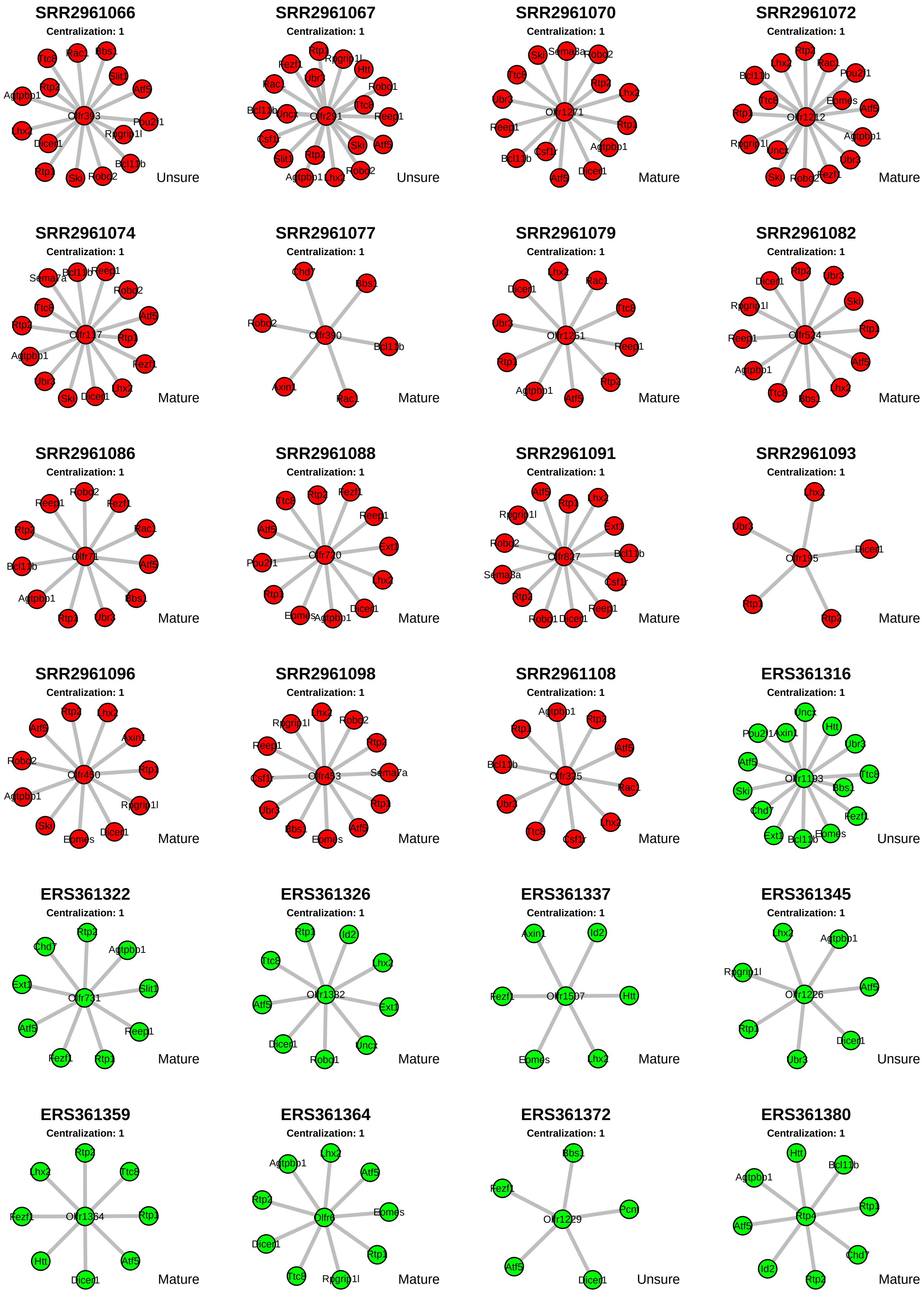
SRR2961066
Centralization: 1
Olfr393
Ttc8 Rac1 Bbs1 Slit1 Atf5 Agtpbp1 Rtp2 Pou2f1 Rpgrip1l Lhx2 Dicer1 Bcl11b Rtp1 Ski Robo2
Unsure
SRR2961067
Centralization: 1
Olfr291
Rtp1 Rpgrip1l Fezf1 Htt Ubr3 Rac1 Robo1 Ttc8 Bcl11b Uncx Reep1 Csf1r Ski Atf5 Slit1 Rtp2 Robo2 Agtpbp1 Lhx2
Unsure
SRR2961070
Centralization: 1
Olfr1271
Ski Sema3a Robo2 Ttc8 Rtp2 Lhx2 Ubr3 Rtp1 Reep1 Agtpbp1 Bcl11b Csf1r Dicer1 Atf5
Mature
SRR2961072
Centralization: 1
Olfr1212
Rtp2 Lhx2 Rac1 Pou2f1 Bcl11b Ttc8 Eomes Atf5 Rtp1 Agtpbp1 Rpgrip1l Uncx Ubr3 Ski Robo2 Fezf1
Mature
SRR2961074
Centralization: 1
Olfr117
Sema7a Bcl11b Reep1 Robo2 Ttc8 Atf5 Rtp2 Rtp1 Agtpbp1 Fezf1 Ubr3 Lhx2 Ski Dicer1
Mature
SRR2961077
Centralization: 1
Olfr390
Chd7 Bbs1 Robo2 Bcl11b Axin1 Rac1
Mature
SRR2961079
Centralization: 1
Olfr1251
Lhx2 Dicer1 Rac1 Ttc8 Ubr3 Reep1 Rtp1 Rtp2 Agtpbp1 Atf5
Mature
SRR2961082
Centralization: 1
Olfr524
Rtp2 Dicer1 Ubr3 Ski Rpgrip1l Rtp1 Reep1 Atf5 Agtpbp1 Ttc8 Bbs1 Lhx2
Mature
SRR2961086
Centralization: 1
Olfr71
Robo2 Reep1 Fezf1 Rtp2 Rac1 Bcl11b Atf5 Agtpbp1 Bbs1 Rtp1 Ubr3
Mature
SRR2961088
Centralization: 1
Olfr720
Ttc8 Rtp2 Fezf1 Reep1 Atf5 Ext1 Pou2f1 Lhx2 Rtp1 Dicer1 Eomes Agtpbp1
Mature
SRR2961091
Centralization: 1
Olfr827
Atf5 Rtp1 Lhx2 Rpgrip1l Ext1 Robo2 Bcl11b Sema3a Csf1r Rtp2 Reep1 Robo1 Dicer1
Mature
SRR2961093
Centralization: 1
Olfr195
Lhx2 Ubr3 Dicer1 Rtp1 Rtp2
Mature
SRR2961096
Centralization: 1
Olfr450
Rtp2 Lhx2 Atf5 Axin1 Robo2 Rtp1 Agtpbp1 Rpgrip1l Ski Dicer1 Eomes
Mature
SRR2961098
Centralization: 1
Olfr453
Lhx2 Rpgrip1l Robo2 Reep1 Rtp2 Csf1r Sema7a Ubr3 Rtp1 Bbs1 Atf5 Eomes
Mature
SRR2961108
Centralization: 1
Olfr325
Agtpbp1 Rtp1 Rtp2 Atf5 Bcl11b Rac1 Ubr3 Lhx2 Ttc8 Csf1r
Mature
ERS361316
Centralization: 1
Olfr1193
Uncx Htt Pou2f1 Axin1 Ubr3 Atf5 Ttc8 Ski Bbs1 Chd7 Fezf1 Ext1 Bcl11b Eomes
Unsure
ERS361322
Centralization: 1
Olfr731
Rtp2 Chd7 Agtpbp1 Ext1 Slit1 Atf5 Reep1 Fezf1 Rtp1
Mature
ERS361326
Centralization: 1
Olfr1332
Rtp1 Id2 Ttc8 Lhx2 Atf5 Ext1 Dicer1 Uncx Robo1
Mature
ERS361337
Centralization: 1
Olfr1507
Axin1 Id2 Fezf1 Htt Eomes Lhx2
Mature
ERS361345
Centralization: 1
Olfr1226
Lhx2 Agtpbp1 Rpgrip1l Atf5 Rtp1 Dicer1 Ubr3
Unsure
ERS361359
Centralization: 1
Olfr1364
Rtp2 Lhx2 Ttc8 Fezf1 Rtp1 Htt Atf5 Dicer1
Mature
ERS361364
Centralization: 1
Olfr6
Lhx2 Agtpbp1 Atf5 Rtp2 Eomes Dicer1 Rtp1 Ttc8 Rpgrip1l
Mature
ERS361372
Centralization: 1
Olfr1229
Bbs1 Fezf1 Pcnt Atf5 Dicer1
Unsure
ERS361380
Centralization: 1
Rtp4
Htt Bcl11b Agtpbp1 Rtp1 Atf5 Chd7 Id2 Rtp2
Mature

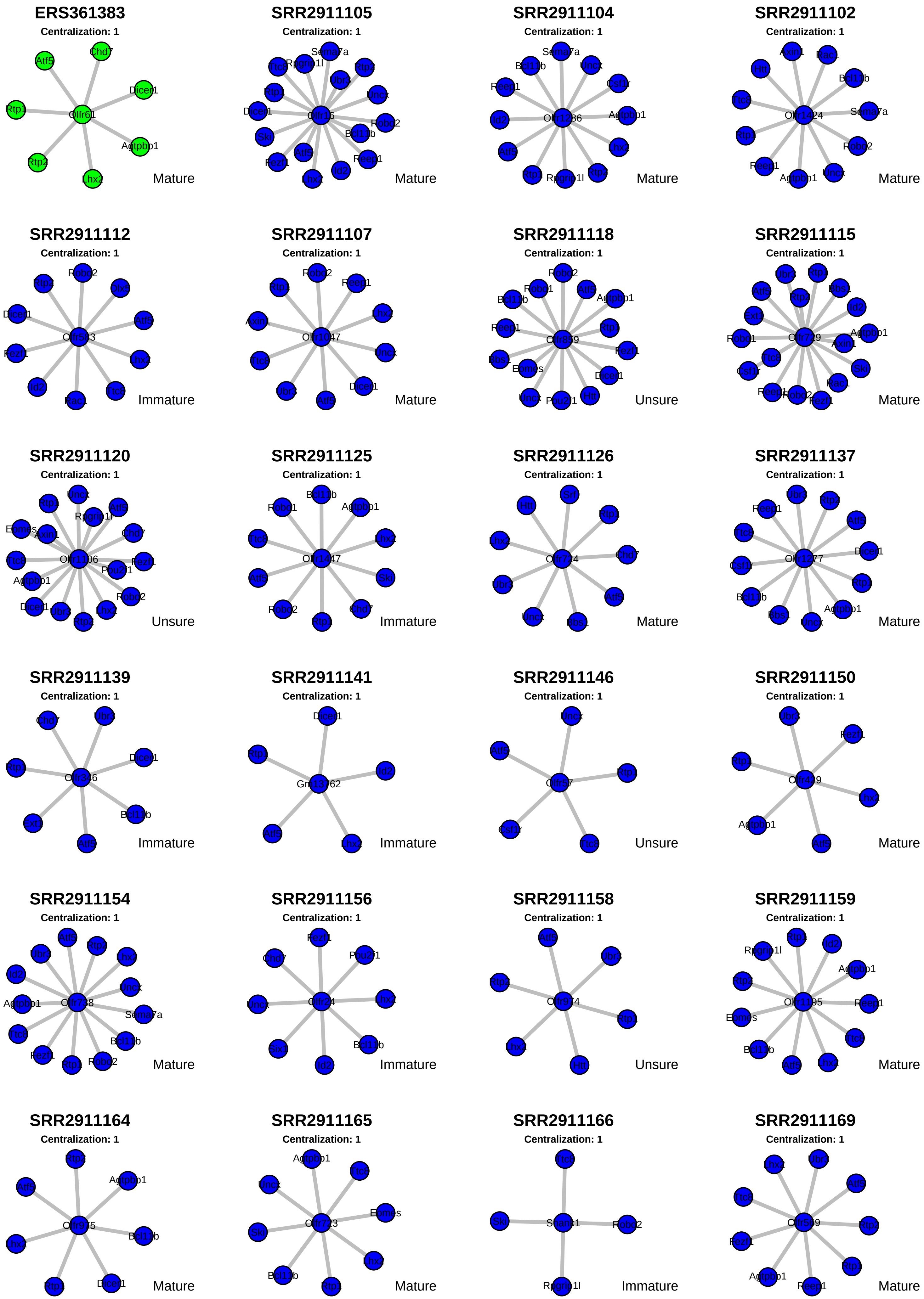
ERS361383
Centralization: 1
Mature
SRR2911105
Centralization: 1
Mature
SRR2911104
Centralization: 1
Mature
SRR2911102
Centralization: 1
Mature
SRR2911112
Centralization: 1
Immature
SRR2911107
Centralization: 1
Mature
SRR2911118
Centralization: 1
Unsure
SRR2911115
Centralization: 1
Mature
SRR2911120
Centralization: 1
Unsure
SRR2911125
Centralization: 1
Immature
SRR2911126
Centralization: 1
Mature
SRR2911137
Centralization: 1
Mature
SRR2911139
Centralization: 1
Immature
SRR2911141
Centralization: 1
Immature
SRR2911146
Centralization: 1
Unsure
SRR2911150
Centralization: 1
Mature
SRR2911154
Centralization: 1
Mature
SRR2911156
Centralization: 1
Immature
SRR2911158
Centralization: 1
Unsure
SRR2911159
Centralization: 1
Mature
SRR2911164
Centralization: 1
Mature
SRR2911165
Centralization: 1
Mature
SRR2911166
Centralization: 1
Immature
SRR2911169
Centralization: 1
Mature

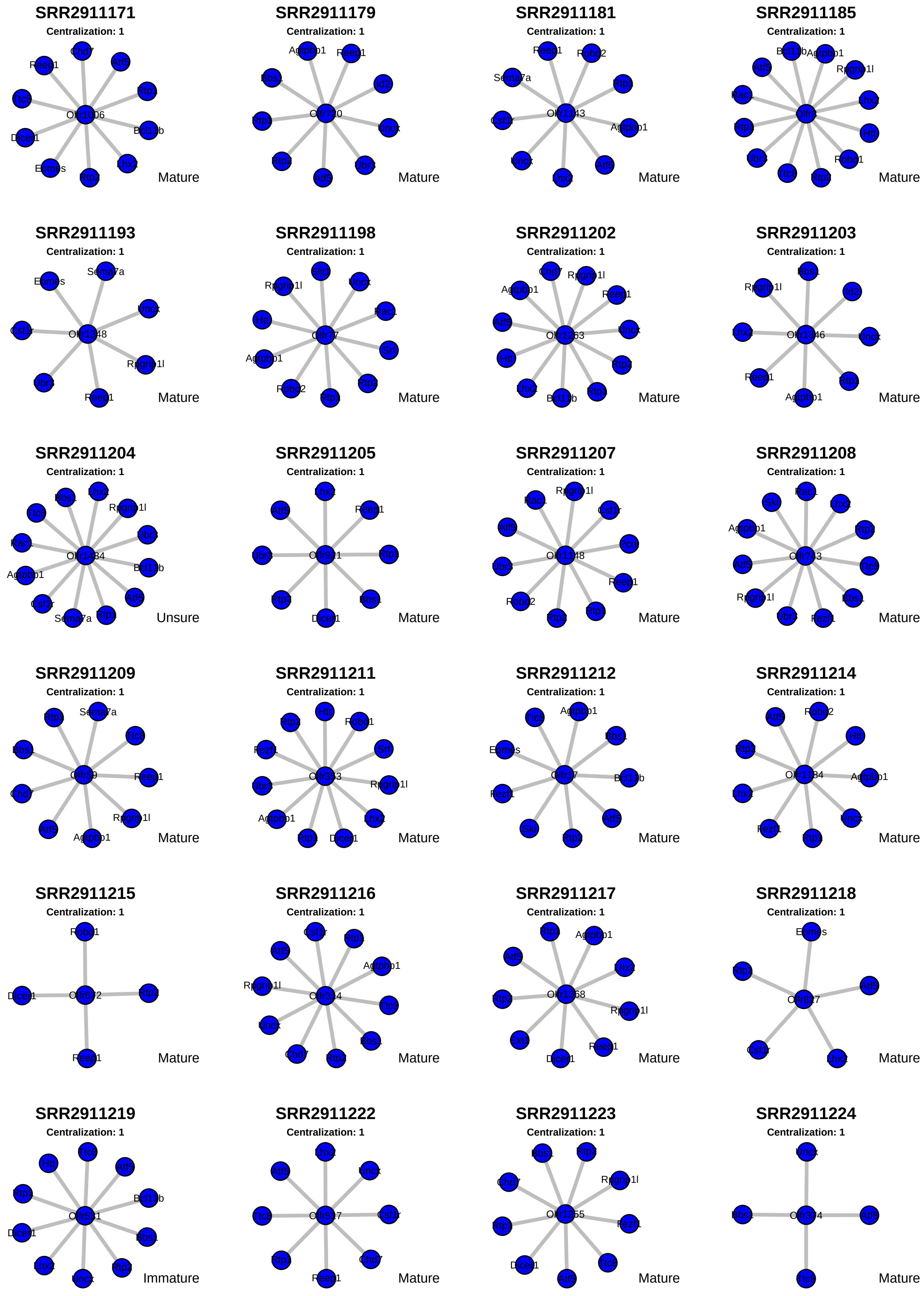
SRR2911171
Centralization: 1
Chd7
Reep1
Atf5
Ttc8
Rtp1
Olfr1006
Dicer1
Bcl11b
Eomes
Lhx2
Rtp2
Mature
SRR2911179
Centralization: 1
Agtpbp1
Reep1
Bbs1
Id2
Rtp1
Olfr720
Uncx
Rtp2
Ubr3
Atf5
Mature
SRR2911181
Centralization: 1
Reep1
Robo2
Sema7a
Rtp1
Csf1r
Olfr1143
Agtpbp1
Uncx
Atf5
Lhx2
Mature
SRR2911185
Centralization: 1
Bcl11b
Agtpbp1
Atf5
Rpgrip1l
Rac1
Lhx2
Olfr3
Rtp1
Htt
Ubr3
Robo1
Ttc8
Rtp2
Mature
SRR2911193
Centralization: 1
Eomes
Sema7a
Uncx
Csf1r
Olfr1348
Rpgrip1l
Ubr3
Reep1
Mature
SRR2911198
Centralization: 1
Slit1
Rpgrip1l
Uncx
Rac1
Htt
Olfr77
Srf
Agtpbp1
Rtp2
Robo2
Rtp1
Mature
SRR2911202
Centralization: 1
Chd7
Rpgrip1l
Agtpbp1
Reep1
Atf5
Uncx
Olfr1263
Htt
Rtp1
Lhx2
Rtp2
Bcl11b
Mature
SRR2911203
Centralization: 1
Bbs1
Rpgrip1l
Id2
Lhx2
Olfr1346
Uncx
Reep1
Rtp1
Agtpbp1
Mature
SRR2911204
Centralization: 1
Bbs1
Lhx2
Rpgrip1l
Ttc8
Ubr3
Rac1
Olfr1434
Bcl11b
Agtpbp1
Atf5
Csf1r
Sema7a
Rtp1
Unsure
SRR2911205
Centralization: 1
Lhx2
Atf5
Reep1
Ubr3
Olfr921
Rtp1
Rtp2
Bbs1
Dicer1
Mature
SRR2911207
Centralization: 1
Rpgrip1l
Rac1
Csf1r
Atf5
Pcnt
Ubr3
Olfr1148
Reep1
Robo2
Rtp2
Rtp1
Mature
SRR2911208
Centralization: 1
Rac1
Ski
Lhx2
Agtpbp1
Rtp1
Atf5
Olfr743
Ttc8
Rpgrip1l
Bbs1
Ubr3
Fezf1
Mature
SRR2911209
Centralization: 1
Rtp1
Sema7a
Ttc8
Bbs1
Olfr59
Reep1
Chd7
Rpgrip1l
Atf5
Agtpbp1
Mature
SRR2911211
Centralization: 1
Htt
Rtp2
Robo1
Fezf1
Srf
Ubr3
Olfr353
Rpgrip1l
Agtpbp1
Lhx2
Rtp1
Dicer1
Mature
SRR2911212
Centralization: 1
Ttc8
Agtpbp1
Bbs1
Eomes
Olfr17
Bcl11b
Fezf1
Atf5
Ski
Rtp2
Mature
SRR2911214
Centralization: 1
Atf5
Robo2
Htt
Rtp2
Olfr1184
Agtpbp1
Lhx2
Uncx
Fezf1
Rtp1
Mature
SRR2911215
Centralization: 1
Robo1
Dicer1
Olfr672
Rtp2
Reep1
Mature
SRR2911216
Centralization: 1
Csf1r
Rtp1
Atf5
Agtpbp1
Rpgrip1l
Olfr314
Ttc8
Uncx
Bbs1
Chd7
Rtp2
Mature
SRR2911217
Centralization: 1
Rtp1
Agtpbp1
Atf5
Lhx2
Rtp2
Olfr1368
Rpgrip1l
Ext1
Reep1
Dicer1
Mature
SRR2911218
Centralization: 1
Eomes
Rtp1
Atf5
Olfr827
Csf1r
Lhx2
Mature
SRR2911219
Centralization: 1
Ttc8
Htt
Atf5
Rtp1
Bcl11b
Olfr531
Dicer1
Bbs1
Lhx2
Uncx
Rtp2
Immature
SRR2911222
Centralization: 1
Lhx2
Atf5
Uncx
Ttc8
Olfr517
Csf1r
Rtp1
Chd7
Reep1
Mature
SRR2911223
Centralization: 1
Bbs1
Rtp1
Chd7
Rpgrip1l
Olfr1255
Rtp2
Fezf1
Dicer1
Ttc8
Atf5
Mature
SRR2911224
Centralization: 1
Uncx
Bbs1
Olfr374
Atf5
Ttc8
Mature

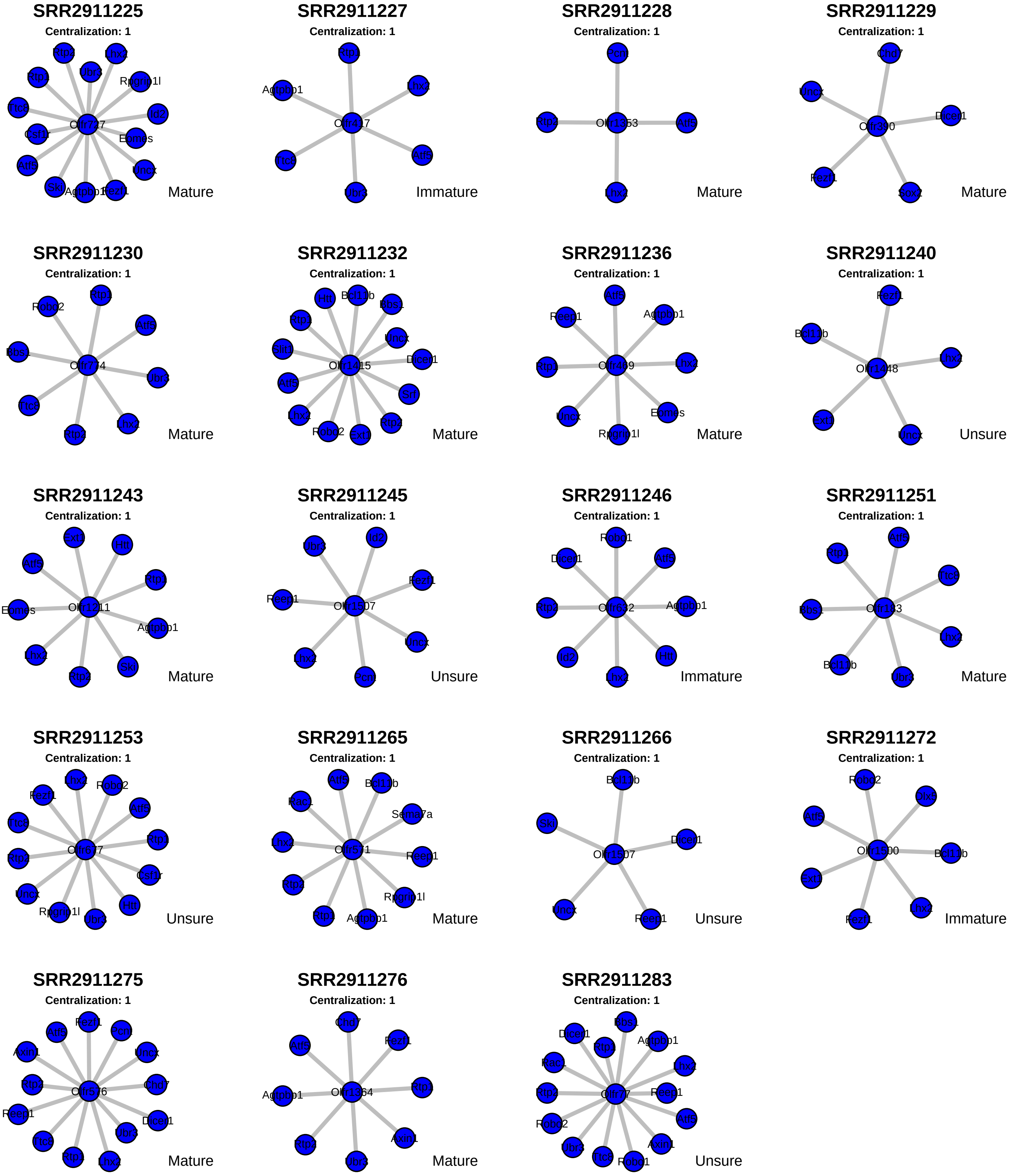

SRR2911225
Centralization: 1
Olfr727
Mature
SRR2911227
Centralization: 1
Olfr417
Immature
SRR2911228
Centralization: 1
Olfr1353
Mature
SRR2911229
Centralization: 1
Olfr390
Mature
SRR2911230
Centralization: 1
Olfr774
Mature
SRR2911232
Centralization: 1
Olfr1415
Mature
SRR2911236
Centralization: 1
Olfr469
Mature
SRR2911240
Centralization: 1
Olfr1448
Unsure
SRR2911243
Centralization: 1
Olfr1211
Mature
SRR2911245
Centralization: 1
Olfr1507
Unsure
SRR2911246
Centralization: 1
Olfr632
Immature
SRR2911251
Centralization: 1
Olfr183
Mature
SRR2911253
Centralization: 1
Olfr677
Unsure
SRR2911265
Centralization: 1
Olfr571
Mature
SRR2911266
Centralization: 1
Olfr1507
Unsure
SRR2911272
Centralization: 1
Olfr1500
Immature
SRR2911275
Centralization: 1
Olfr576
Mature
SRR2911276
Centralization: 1
Olfr1364
Mature
SRR2911283
Centralization: 1
Olfr77
Unsure
